# Supplementary material for: Gut microbiota produces biofilm-associated amyloids with potential for neurodegeneration
Source: Nat Commun. 2024 May 16;15:4150. doi: 10.1038/s41467-024-48309-x (PMC11099085; doi:10.1038/s41467-024-48309-x)
Supplement: Supplementary file 1 — Supplementary Information [file 41467_2024_48309_MOESM1_ESM.pdf]

SUPPLEMENTAL INFORMATION

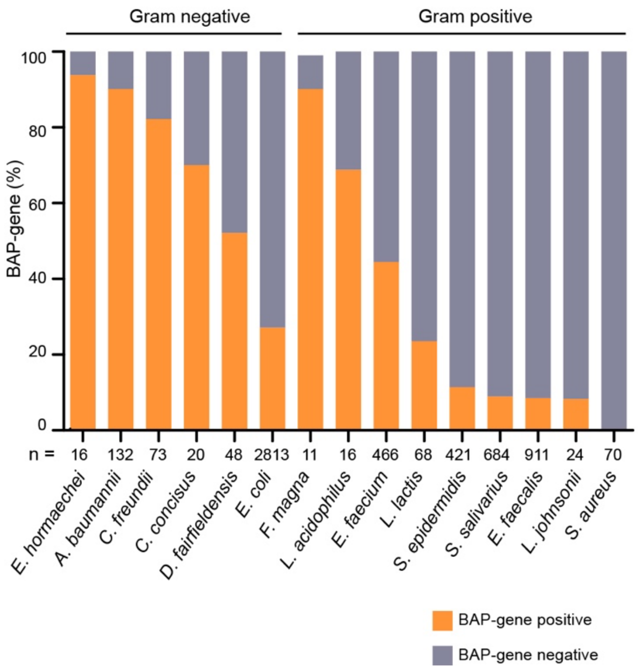

Figure S1. Frequency of presence (orange)/absence (grey) of BAP genes. The presence (orange)/absence (grey) binary matrix of the bacterial species was queried with the corresponding BAP-like gene reference. N indicates the number of genomes analyzed. Source data are provided as a Source Data file.

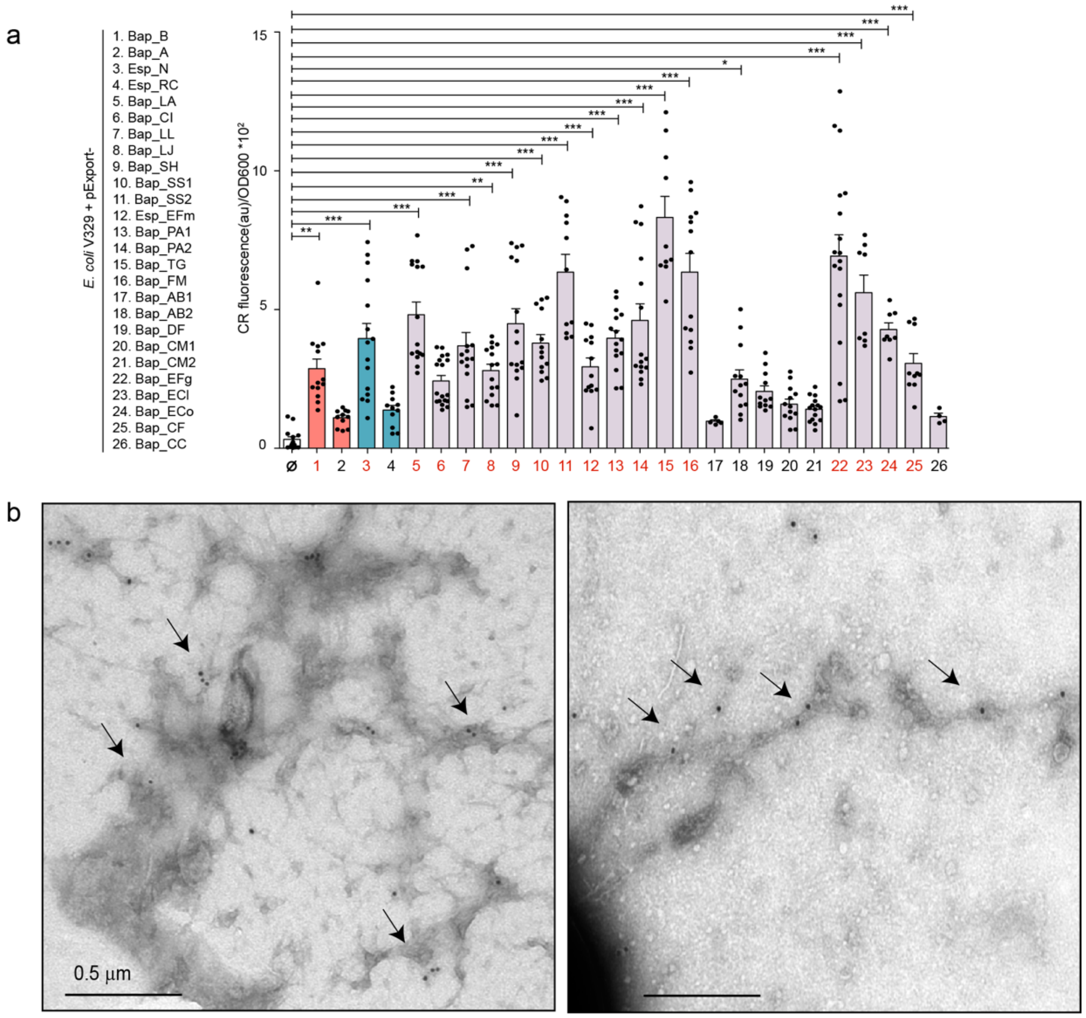

Figure S2. BAP domains form amyloid-like structures. **a** Quantification of CR binding of *E. coli* cells that export the predicted BAP amyloidogenic domains grown at 37ºC. CR fluorescence (ex, 525 nm; em, 625 nm) and bacterial growth (OD_{600}) were obtained at BioTek Synergy H1 microplate reader. CR fluorescence/OD_{600} ratio was calculated at the endpoint (16 h) to normalize CR fluorescence values according to bacterial growth. *E. coli* expressing Bap_B and Esp_N were used as positive controls (numbers 1 and 3 respectively). *E. coli* expressing Bap_A and Esp_RC (numbers 2 and 4 respectively) were used as negative controls. CR binding of *E. coli* strains expressing BAP-derived domains were compared to CR binding of *E. coli* without the pExport plasmid (Ø). *N*=18 for *E. coli* expressing Bap_CI, Bap_EFg; *N*=15 EspN, Bap_LA, Bap_LL, Bap_LJ, BVap_SH, Bap_PA1, Bap_PA2, Bap_CM2; *N*=13 Bap_B, Bap_SS1, Bap_EFm, Bap_FM, Bap_AB2, Bap_DF, Bap_CM1; *N*=11 Bap_A, Esp_C, Bap_SS1; *N*=10 Bap_TG, Bap_CF; *N*=8 Bap_CI, Bap_Eco; *N*=5 Bap_AB1; *N*=4 Bap_CC. Statistically significant differences were determined using one way ANOVA and Bonferroni's multiple comparison test $*p < 0.05$, $**p < 0.01$, $***p < 0.001$. **b** Immunogold-labeled samples of fibers formed by *E. coli* expressing Bap_SS2 (left panel) and Bap_CI (right panel) using anti-His antibodies. Bap_SS2 and Bap_CI were cloned into the pExport plasmid that allows the expression of the epitope of interest fused to the first 42 residues of the signal sequence of CsgA (ssCsgA) and the 6xHistidine tag. Bars represent 0.5 µm. Source data are provided as a Source Data file.

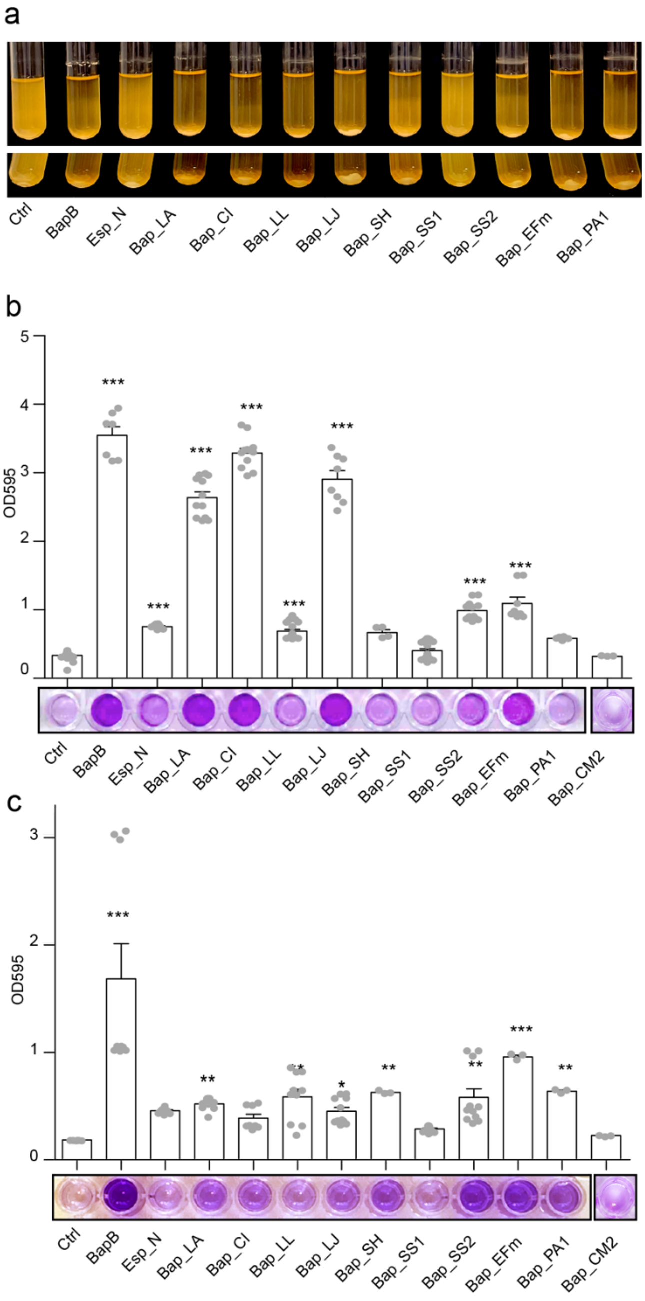

Figure S3. Expression of BAP N-terminal domains is sufficient to mediate biofilm phenotype in a heterologous strain. a Bacterial clumping of *S. aureus* Δbap mutant expressing chimeric N-terminal domains of BAPs. Images of the bottom part of the tubes are shown (bottom panel). Cultures were grown overnight in TSBg supplemented with 1 μM CdCl2 under shaken conditions (200 rpm) at 37 °C. **b** Biofilm formation of *S. aureus* Δbap cells that express the BAP N-terminal domains. For biofilm formation, bacteria were cultured overnight in TSBg supplemented with cadmium 1 μM CdCl2 at 37 °C in microtiter plates under static conditions. **c** Biofilm formation without cadmium supplementation. As positive controls, Bap_B and Esp_N chimeric protein were used. As negative control, a chimeric protein using the non-amyloid domain Bap_CM2 was constructed. Data are the result of at least three replicates of two independent experiments. Data are shown as means, and error bars are shown as the SD of means. Statistically significant differences were determined using non parametric Kruskal Wallis test $*p < 0.05$, $**p < 0.01$, $***p < 0.001$. Source data are provided as a Source Data file.

A

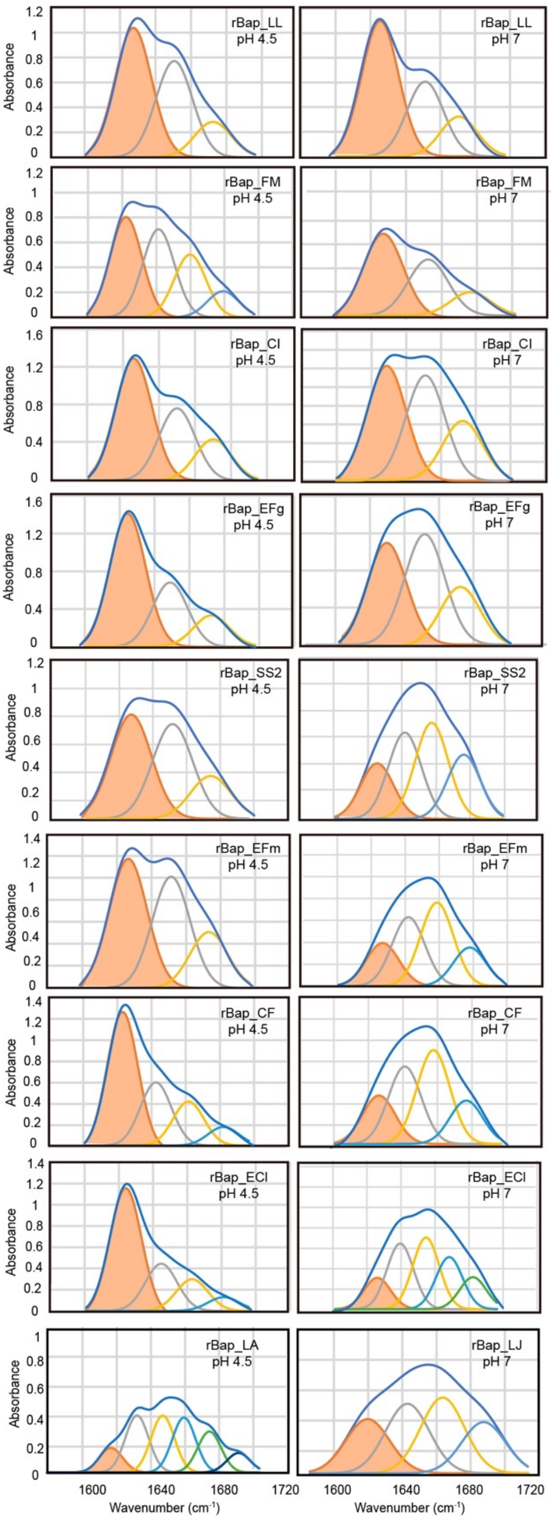

B

rBap_LL pH 4.5

Band (cm^{-1})	% Area
1625	27.7
1650	20.6
1673	7.8

rBap_LL pH 7

Band (cm^{-1})	% Area
1625	29.1
1650	16
1673	8.5

rBap_FM pH 4.5

Band (cm^{-1})	% Area
1627	19.1
1652	16.7
1673	11.9
1683	4.9

rBap_FM pH 7

Band (cm^{-1})	% Area
1624.8	19.38
1649.4	13
1672.6	5.4

rBap_CI pH 4.5

Band (cm^{-1})	% Area
1625	36.2
1650	20.2
1673	11.5

rBap_CI pH 7

Band (cm^{-1})	% Area
1628	34.5
1652	31.6
1673	17.7

rBap_EFg pH 4.5

Band (cm^{-1})	% Area
1625	36.2
1650	15.5
1673	8.5

rBap_EFg pH 7

Band (cm^{-1})	% Area
1628	32
1650	34.8
1672	18.7

rBap_SS2 pH 4.5

Band (cm^{-1})	% Area
1627	24.4
1652	22.2
1673	10

rBap_SS2 pH 7

Band (cm^{-1})	% Area
1626	18,4
1641	28,7
1657	31.8
1676	21

rBap_EFm pH 4.5

Band (cm^{-1})	% Area
1627	32.3
1652	27.9
1673	14

rBap_EFm pH 7

Band (cm^{-1})	% Area
1629	9.5
1642	15.2
1658	18.4
1679	8.5

rBap_CF pH 4.5

Band (cm^{-1})	% Area
1623	24.5
1642	11.6
1661	8.1
1682	3.4

rBap_CF pH 7

Band (cm^{-1})	% Area
1627	9.5
1642	15.2
1658	18.4
1679	8.5

rBap_ECl pH 4.5

Band (cm^{-1})	% Area
1624	26.9
1645	10.3
1663	6.9
1682	3

rBap_ECl pH 7

Band (cm^{-1})	% Area
1626	6.22
1639	13
1655	14.18
1669	10.32
1683	6.35

rBap_LA pH 4.5

Band (cm^{-1})	% Area
1629	1.99
1641	4.62
1653	4.61
1663	4.45
1674	3.32
1687	1.58

rBap_LJ pH 7

Band (cm^{-1})	% Area
1626	21.6
1643	27.8
1659	30.2
1677	20.4

C

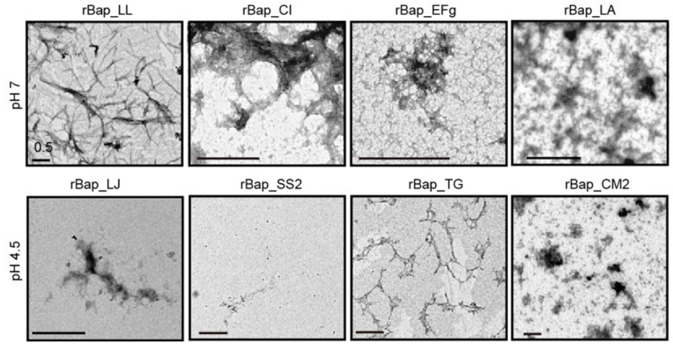

Figure S4. Biophysical characterization of amyloidogenic domains of BAPs. a Deconvolution of the FTIR spectra of aggregates at pH 4.5 and at pH 7. Component bands after Gaussian deconvolution are shown. **b** Percentage of each secondary structure is detailed in the tables. **c** Representative transmission electron micrograph of BAP amyloid fibers after incubation at pH 7 **d** Representative transmission electron micrograph of amorphous aggregates. Source data are provided as a Source Data file.

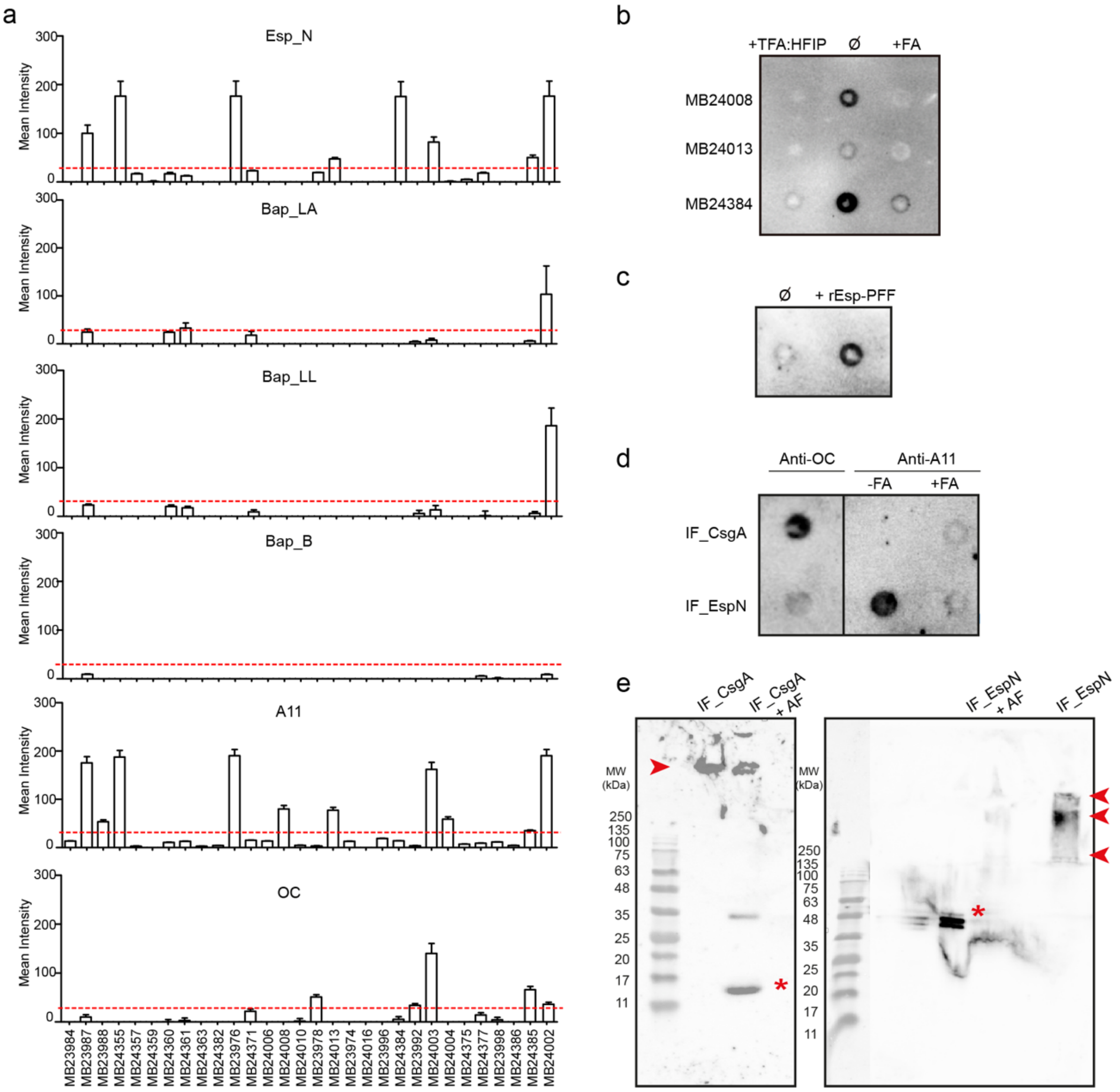

Figure S5. BAP amyloid-like structures are present in the human fecal microbiota. a Quantification of BAP-related amyloids from microbiota. The relative signal intensity of the blots was determined by densitometry using Image Lab Software. GraphPad Prism Version 6 was used for density analysis. **b** Insoluble protein fractions purified from human stool samples were treated with 100% formic acid (FA) or a solution of trifluoroacetic acid/hexafluoroisopropanol (TFA:HFIP) (1:1). After treatment, samples were subjected to vacuum filtration through a 96-well dot blot apparatus with an acetate cellulose membrane. Immunoblotting using anti Esp_N antibodies showed no retained aggregates. **c** Detection of Esp amyloids in stool samples of mice subjected to a broad-spectrum antibiotics treatment to deplete the gut microbiota (Ø). After antibiotics treatment, rEspN_PFF were orally administered for 2 days. The SDS resistant fraction purified from mice faecal samples were subjected to a filter retention assay. Immunoblotting was performed using anti Esp_N antibodies. **d** Detection of CsgA and Esp amyloids purified from the insoluble fraction of *E. coli* VS39 pExport-CsgA (IF_CsgA) and *E. coli* VS39 pExport-EspN (IF_EspN) using the anti-amyloid oligomers (A11) and the anti-amyloid fibrils OC antibodies. Samples were treated (+FA) or not (-FA) with formic acid. **e** Immunodetection of CsgA and Esp in the purified insoluble fraction using anti-His and anti-Esp antibodies, respectively. Mature fibres of CsgA and amyloid oligomers of Esp were marked with arrows. Monomers of CsgA and Esp were marked with asterisks. Source data are provided as a Source Data file.

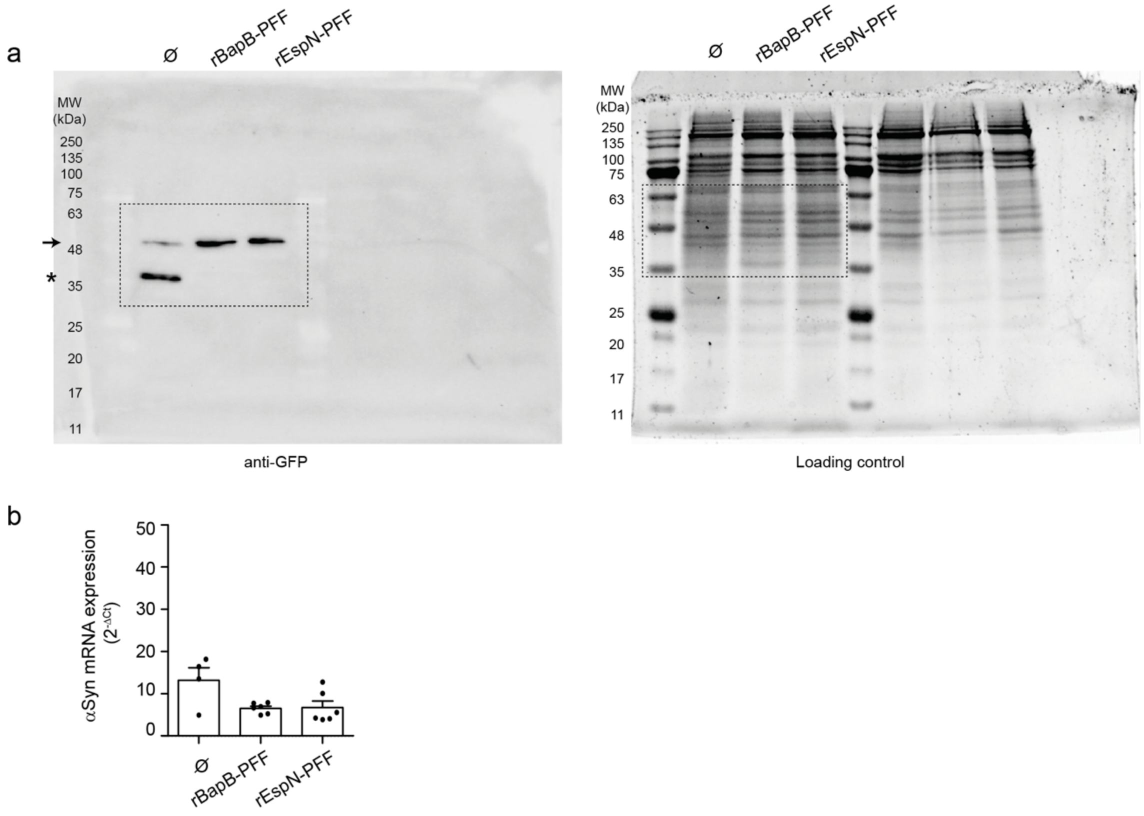

Figure S6. Effect of BAP-derived amyloidogenic domains on α-Syn aggregation in *C. elegans* model of PD. a Uncropped figure 4f. Immunodetection of α-Syn:YFP in the protein fraction of *C. elegans* NL5901 fed *E. coli* expressing Bap_B and Esp_N amyloids. Anti-GFP antibody was used to detect α-Syn. Stained SDS-PAGE was used as loading control. Arrow and * indicate α-Syn monomeric and sub-monomeric forms respectively. **b** qPCR measurement of α-syn mRNA level in day-10 *C. elegans* fed *E. coli* OP50 supplemented with rBapB-PFF and rEspN-PFF. Two biological replicates were performed. Statistically significant differences were determined using non parametric Kruskal Wallis Test. Source data are provided as a Source Data file.

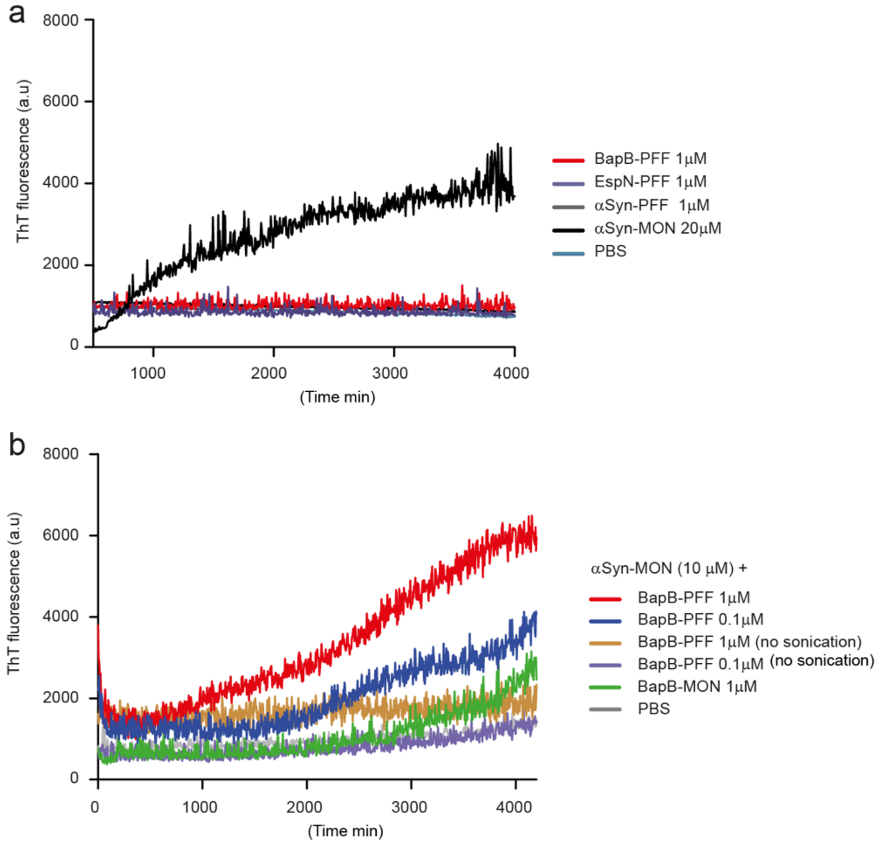

Figure S7. Aggregation kinetics by measuring Th-T fluorescence over time. a Th-T fluorescence of the seeds obtained after sonication (60 minutes) of rBapB-PFF, rEspN-PFF and α-Syn-PFF to show that the seeds alone do not alter Th-T signal. As positive control aggregation kinetic of monomeric α-Syn was shown. **b** Aggregation kinetics of monomeric α-Syn in the absence or presence of the indicated amount of seeds obtained after sonication (60 minutes) of rBapB-PFF, rBapB-PFF nonsonicated or monomeric rBapB (rBapB-MON). Source data are provided as a Source Data file.

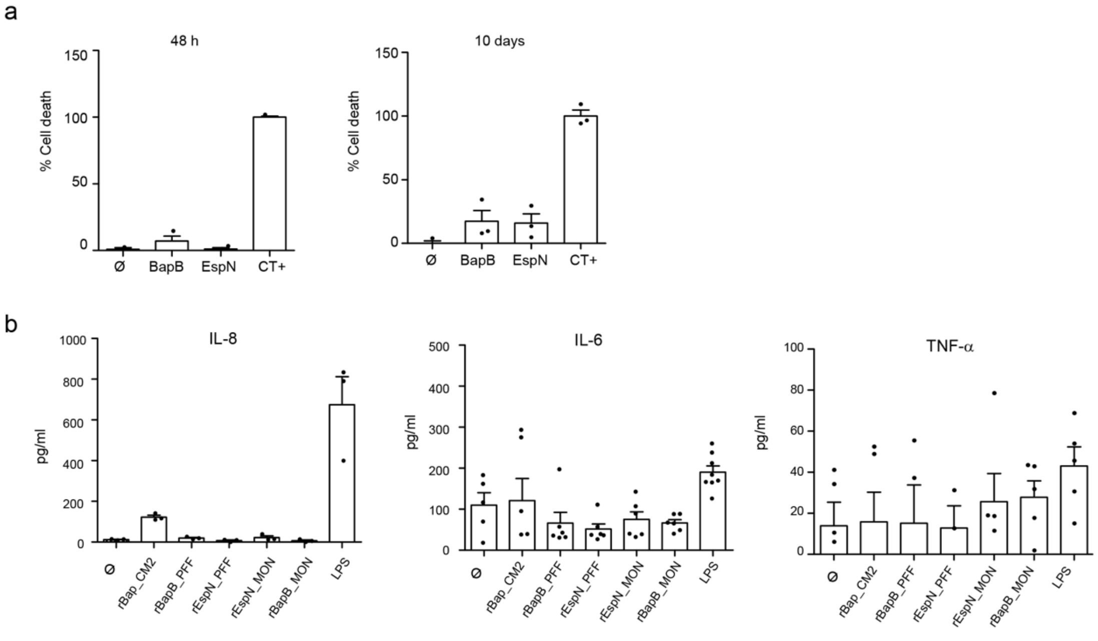

Figure S8: Effect of BAP-amyloids on cell viability and cytokine release. **a** Differentiated SH-SY5Y cells were treated for 2 days with 0.02 µg/µl of rBapB-PFF and rEspN-PFF. LDH assay was used to determine cell viability (% cell death in comparison with the positive control). Data are shown as means, and error bars are shown as the SE of means. (*N*=3**b** Differentiated SH-SY5Y cells were treated for 2 days with 0.02 µg/µl of preformed fibrils (BapB-PFF and EspN-PFF), monomeric proteins (BapB-MON and EspN-MON), the non-amyloid domain Bap_CM2 and LPS (0.05 µg/ml). The concentration of IL-8, IL-6 and TNF-α cytokines in the supernatant was measured by ELISA. IL-8 (*N*=3); IL-6 (*N*=6), TNF-α (*N*=5). Values are expressed in pg/ml Data are represented as means, and error bars are shown as the SE of means. Negative values were not represented in the graphs. Data were analyzed using Kruskal Wallis with Dunn's multiple comparison test. Source data are provided as a Source Data file.

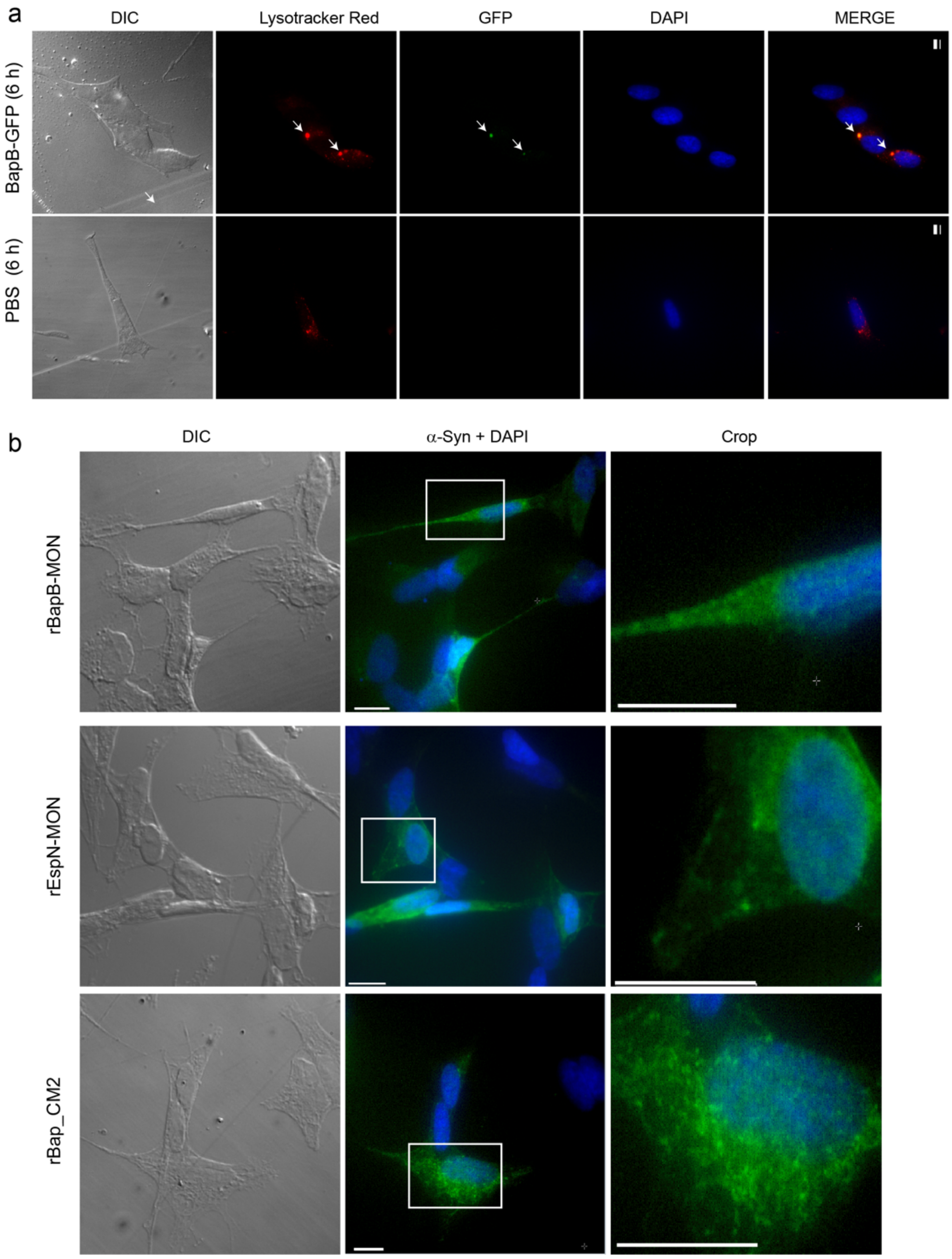

Figure S9. **Effect of BAP amyloids in α-Syn aggregation. a** Intracellular localization of BAP-type amyloids by fluorescence microscopy. To assess the internalization of rBapB:GFP amyloid fibers, differentiated SH-SY5Y cells were seeded on 13 mm circular coverslips in 24-well plates. Cells were treated with 0.02 µg/µl of rBapB-PFF conjugated with GFP for 6 h. The intracellular acidic compartments were stained treating neurons with 0.5 µM of LysoTrackerTM Red (Invitrogen) for 15 min. Cells were washed in PBS, fixed with 4% PFA for 20 min at room temperature. DNA was stained with Hoechst 33342 diluted 1:1000 (v:v). Fixed samples were imaged at 100X with a Leica DMi8 fluorescence microscope and Hamamatsu ORCA Flash 4.0 LT camera. Image processing was performed with Icy software. Two independent experiments were done. Scale bars, 5 µm. **b** Effect of the monomeric proteins rBap_B and rEsp_N (rBapB-MON and rEspN-MON) and the non-amyloid domain rBap_CM2 on α-Syn aggregation. Representative images of immunostaining of α-Syn (green) in SH-SY5Y cells expressing wild-type α-Syn with a C-terminal HA tag treated with 0.02 µg/µl of rBapB-MON, rEspN-MON and rBap_CM2. Scale bars,10 µm. Higher magnifications of the highlighted regions are shown.

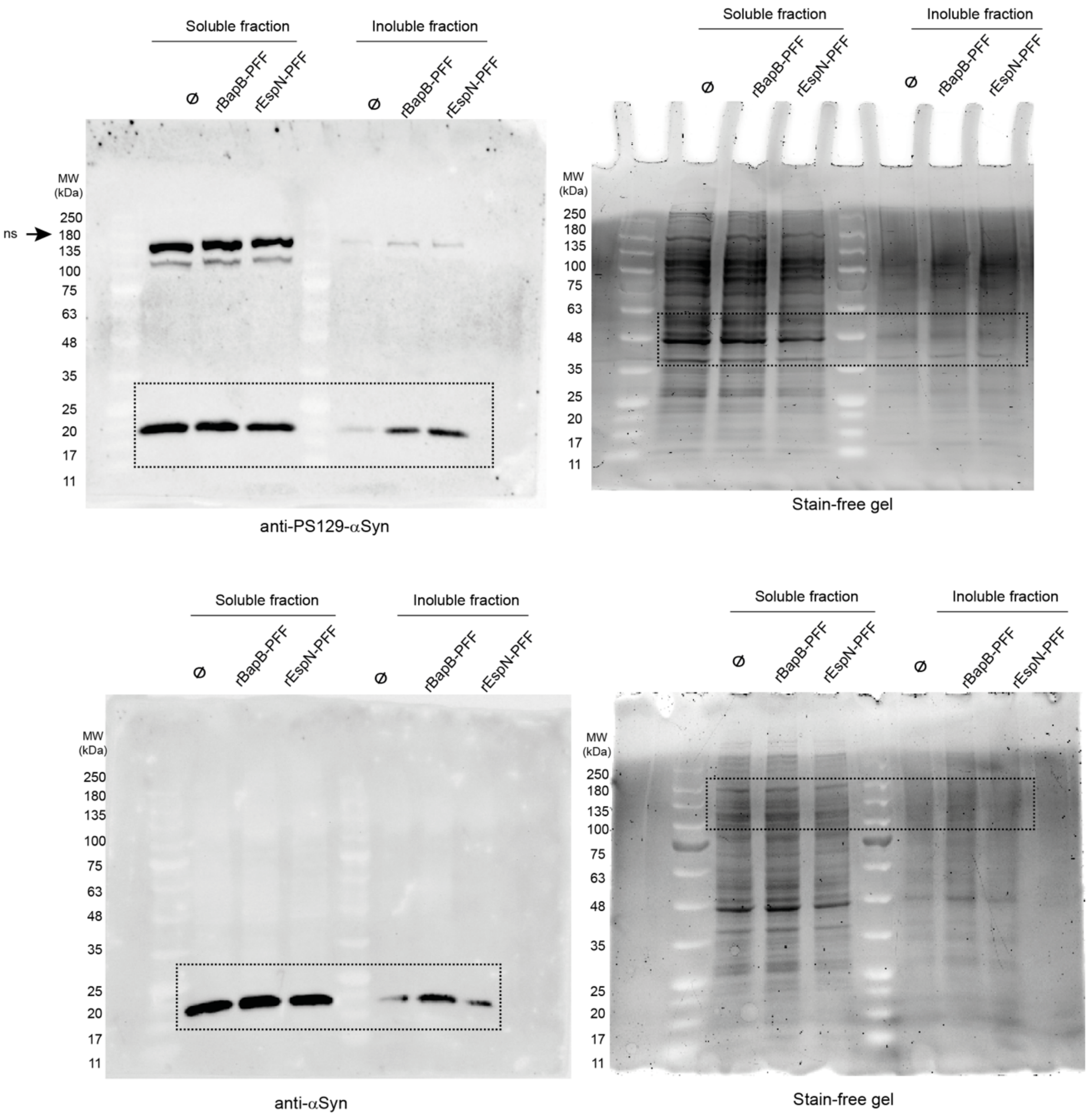

Figure S10. BAP-derived amyloids increase α-Syn levels. Uncropped figure 5i. Cell lysates of SH-SY5Y cells incubated with 0.02 μg/μl of rBap-PFF and rEspN-PFF were fractionated into 1% Triton X-100-soluble and -insoluble fractions, followed by immunoblotting with anti-PS129-α-Syn (ab51253) antibody or anti-α-Syn (LB509). Stain-free gels are shown as a loading control. Dot boxes were cropped to shown in figure 5i. ns: unknown protein stained with anti-PS129-α-Syn (ab51253) antibody.

a

Cerebral structure / Injection site	Striatum	
Time	24h	72h
Motor cortex		
Visual cortex		
Somatosensory cortex	++	+++
Hippocampus		
Enthorrinal cortex		++
Olfactory areas		
Striatum	+++	+++
Amygdala	+++	++
Thalamus	+	+
Hypothalamus	++	++
SNpc		

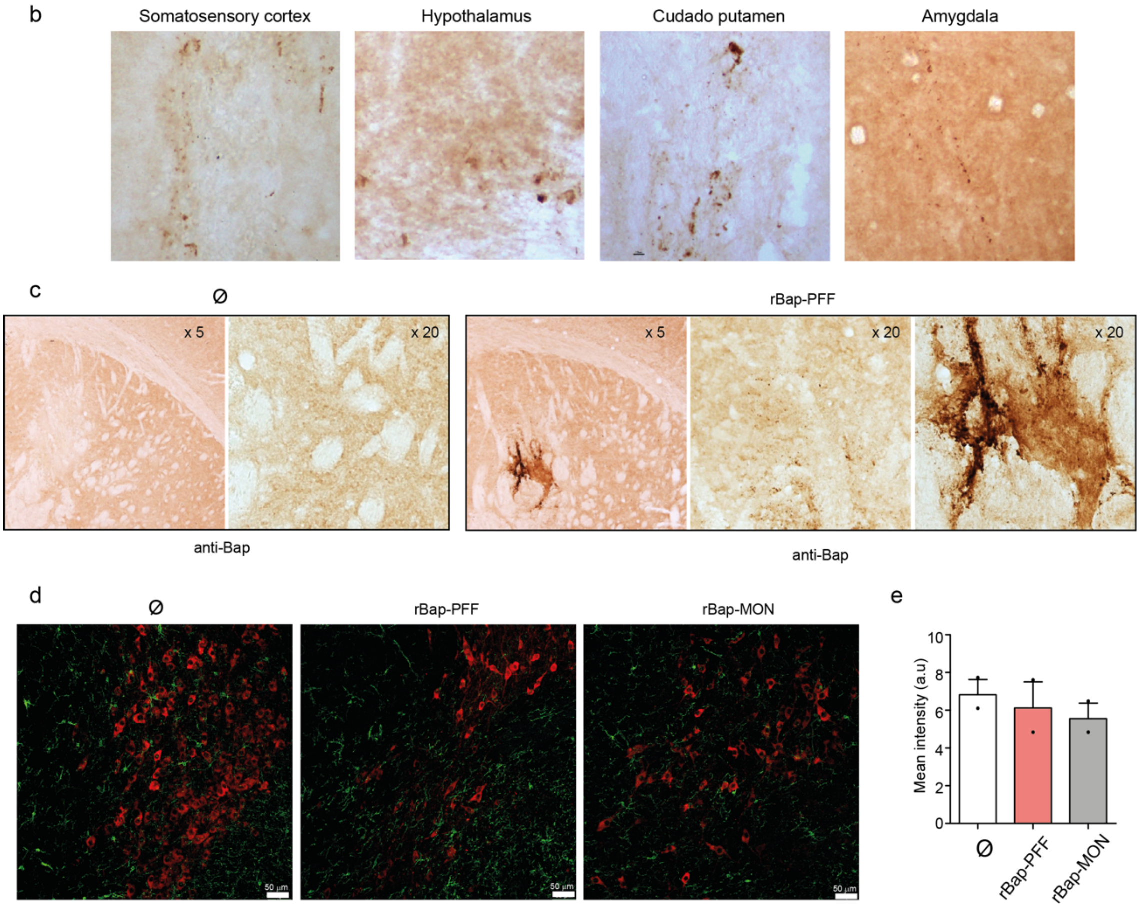

Figure S11. Analysis of the presence of rBap-PFF at different brain regions. a Summary table of the presence of rBap-PFF on different brain structures. C57BL6/C3H F1 mice (*N*=10) received an injection of sonicated rBap-PFF (10 µg/mice) into the dorsal striatum by stereotaxic injection. Mice were sacrificed at 24 h or 72 h post injection and the presence of Bap aggregates were determined by immunostaining using anti-Bap antibodies. **b** rBap-PFF immunostaining in different brain sections. **c** Control for anti-Bap antibodies specificity. Mice that received PBS (Ø) by stereotaxic injection did not show a Bap-specific staining, while mice that received rBap-PFF showed clear Bap-specific staining. **d** Representative images showing immunostaining of IBA-1 microglia/macrophages on mid-brain sections of mice treated with rBap-PFF and rBap-MON. TH-positive neurons are shown in red. **e** Graphs showing quantification of the fluorescence intensity corresponding to Iba-1 (*N*=2). Data are represented as means, and error bars are shown as the SE of means. Data were analyzed using two tailed Mann–Whitney test. Red bards: mice intrastriatally injected with rBapB-PFF. Grey bars: mice intrastriatally injected with Bap-MON. White bards: mice intrastriatally injected with PBS. Source data are provided as a Source Data file.

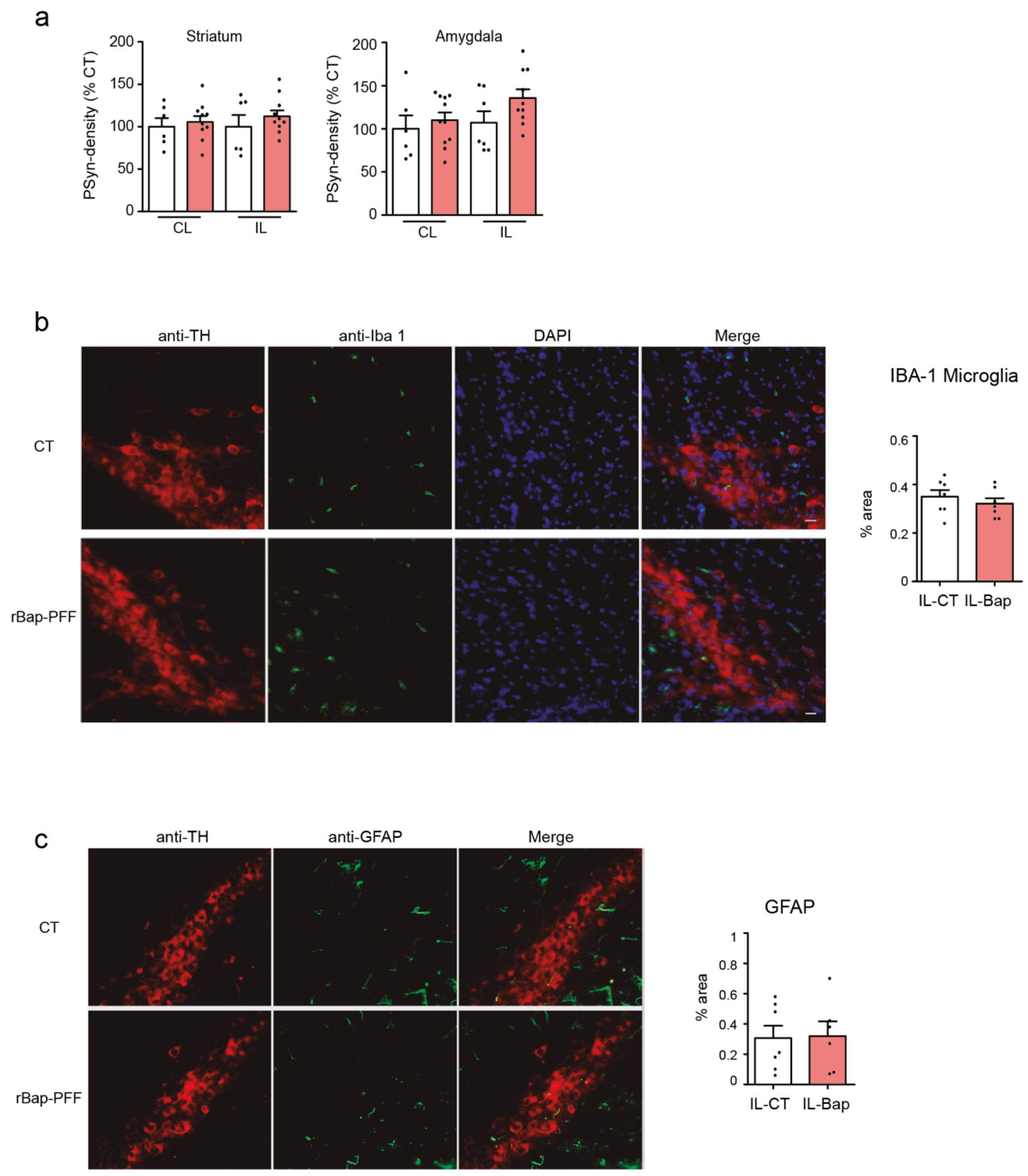

Figure S12. Effect of BAP amyloid fibers in phosphorylated α-Syn and the immune response. a Quantification of Ser129 phospho-α-Syn (P-Syn) densitometry in the contralateral (CL) and ipsilateral (IL) of striatum and amigdala of mice treated with rBapB-PFF amyloids (N=10) (red bars) and control mice (N=6) (white bars). Representative images showing immunostaining of Iba-1 (**b)** microglia/macrophages and GFAP (**c**) on mid-brain sections of mice treated with rBapB-PFF amyloids and control mice (CT). TH-positive neurons are shown in red. Graphs showing quantification the % of the area occupied by positive Iba-1 and GFAP (N=7). For all the panels data are shown as means, and error bars are shown as the SE of means. Statistically significant differences were determined using two-tailed Mann–Whitney test. Source data are provided as a Source Data file.

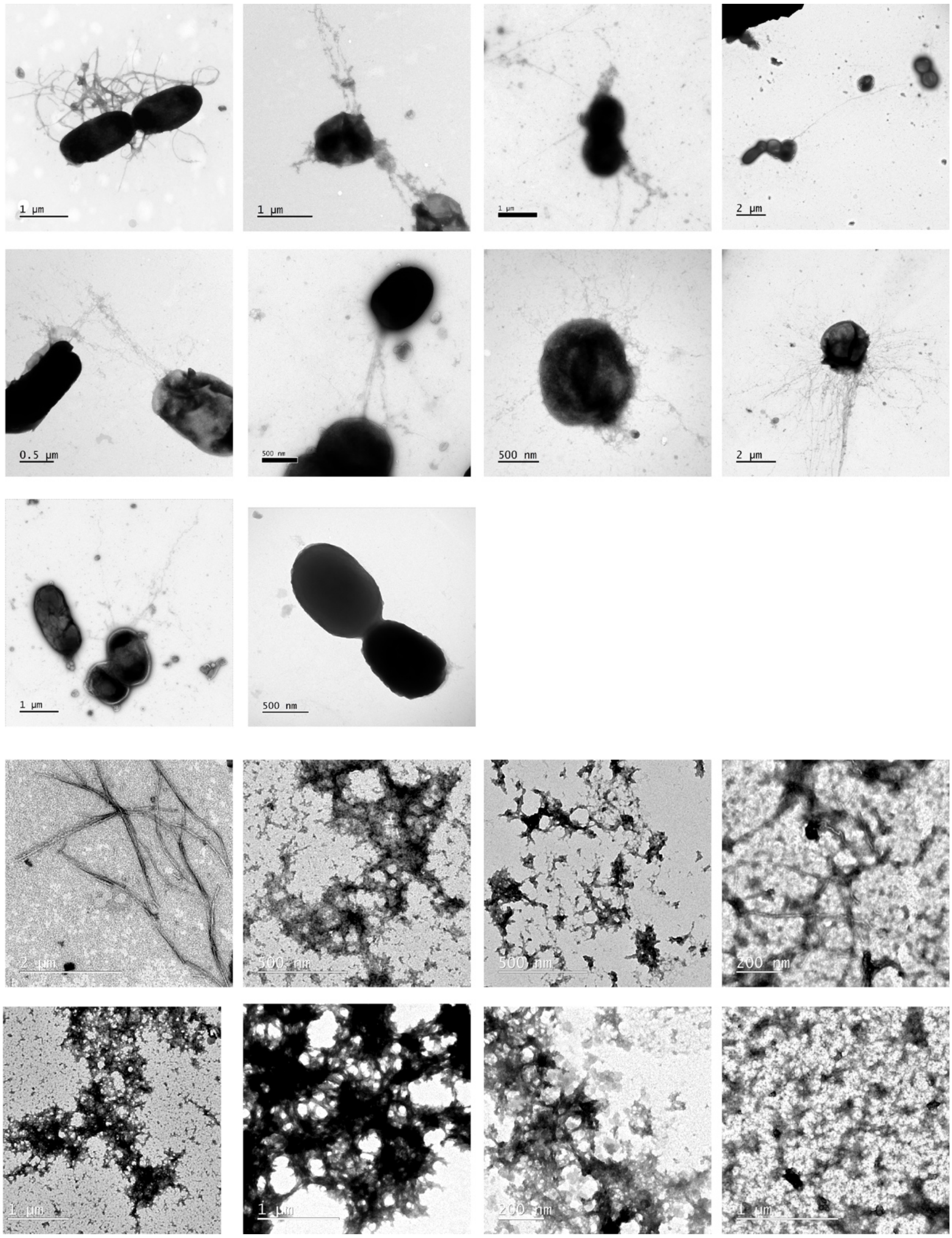

Figure S13. Uncropped and unprocessed transmission electron micrographs

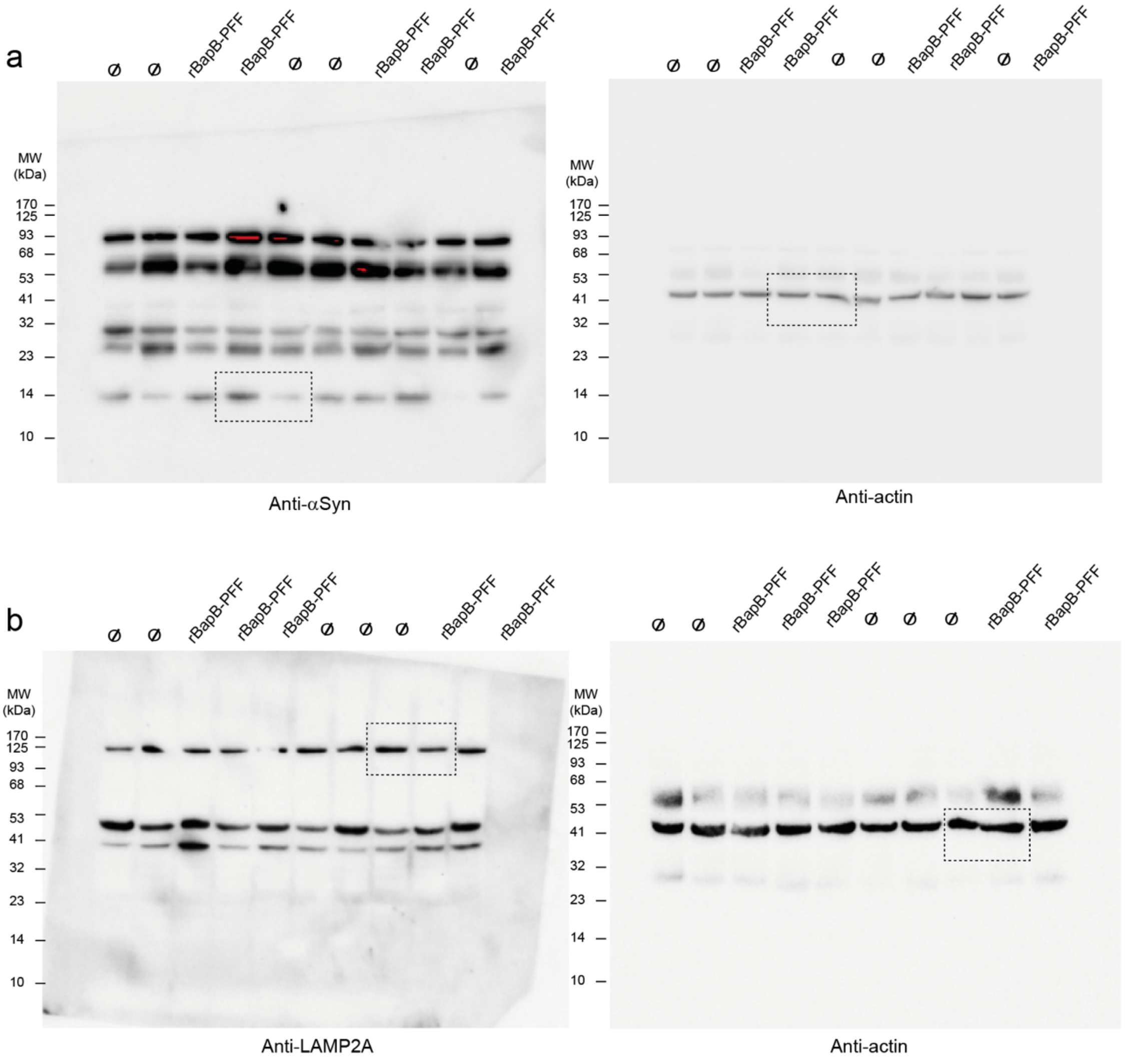

Figure S14. Effect of rBapB-PFF in the expression of αSyn and LAMP-2A. Uncropped figure 6j. Western-blot of cell lysates from midbrain of mice after injection of rBap-PFF, followed by immunoblotting with anti-α-Syn and anti-actin antibodies (**a**) and anti-LAMP2A and anti-actin antibodies (**b**). Dot boxes were cropped to shown in figure 6j.

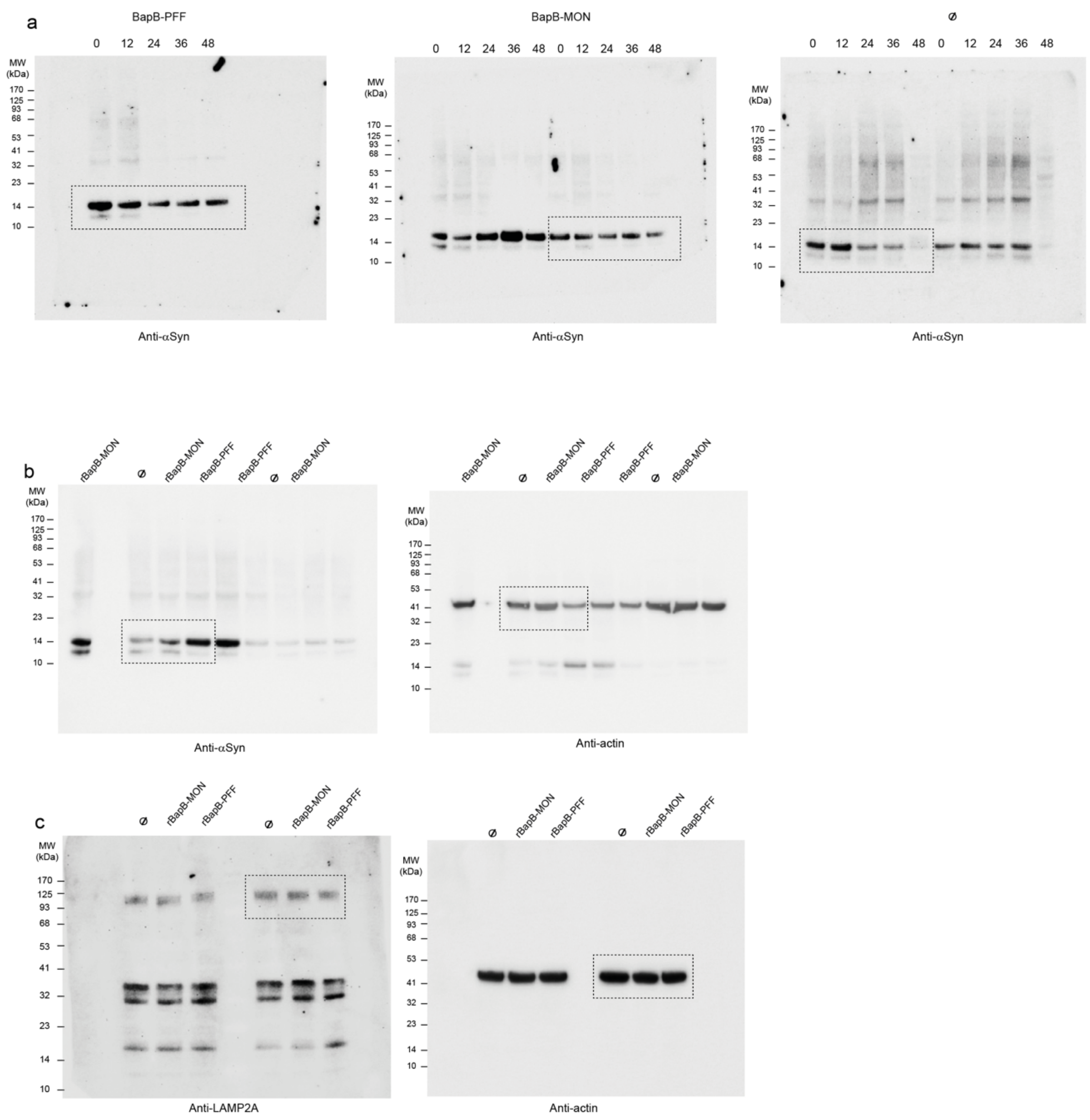

Figure S15. Effect of rBapB-PFF in the expression of αSyn and LAMP-2A. Uncropped figure 6l and 6n. **a** Western-blot analyzing synuclein turnover during 48 hours under in SH-SY5Y cells incubated with rBapB-PFF and rBap-MON. Dot boxes were cropped to shown in figure 6l. Western-blot of SH-SY5Ycell lysates treated with rBapB-PFF and rBapB-MON followed by immunoblotting with anti-α-Syn and anti-actin antibodies (**b**) or anti-LAMP2A and anti-actin antibodies (**c**). Dot boxes were cropped to shown in figure 6n.

Table S1. BAP-proteins

Bacteria	Accession nº	Length Amino acids	Nº repeats	% Identity Bap	Signal peptide					
					Other	SP (Sec/SPI)	LP SP (Sec/SPII)	TAT SP (Tat/SPI)	TAT LP SP (Tat/SPII)	Pilin-like SP (Sec/SP III)
Staphylococcus aureus	AAK38834.2	2276	13C	-	0.0008	0.9984	0.0002	0.0002	0.0002	0.0002
Clostridium indolis	VDG65910	2313	2A, 7C	58	0.0025	0.9963	0.0005	0.0003	0.0002	0.0002
Enterococcus faecalis	VTT42696.1	1953	3AB, 7C	35	0.0965	0.9006	0.002	0.0004	0.0003	0.0003
Terribacillus goriensis	AIF67599.1	2839	3B, 8C, 11D	36	0.7001	0.2903	0.0069	0.0009	0.0008	0.0001
Chania multitudinisentens	AHG22648	6495	4A, 5B	36	1.0001	0	0	0	0	0
Streptococcus salivarius	MTR00538.1	1720	10C	29	0.0182	0.9729	0.0079	0.0004	0.0003	0.0003
Lelliottia amnigena	ANG93841	3726	-	29	1	0.0001	0	0	0	0
Lactococcus lactis IO-1	BAL51295.1	1372	5C	28	0.3723	0.6067	0.0112	0.0066	0.0022	0.0001
Escherichia fergusonii	CAQ90958.1	3063	-	28	1	0	0	0	0	0
Enterobacter 368	ABP61783.1	3552	-	28	1.0001	0	0	0	0	0
Cedecea neteri SSMD04	AIR06275.1	4190	-	28	1	0	0	0	0	0
Enterobacter hormaechei subsp. xiangfangensis	AJB81761.1	6017	5B	28	1.0001	0	0	0	0	0
Acinetobacter baumannii	CAM85746.1	7640	20B, 28CD	27	1	0	0	0	0	0
Enterococcus faecium	AGS76773	1732	3AB, 6C	27	0.0242	0.9737	0.0013	0.0004	0.0002	0.0002
Salmonella enterica subsp. enterica	NP_457158	3624	-	27	0.9994	0.0006	0	0	0	0
Desulfovibrio fairfieldensis	AMD89374	6508	18B, 17C	26	1.0001	0	0	0	0	0
Escherichia coli O145:H28	AHG13161.1	5662	6B	25	0.9999	0.0002	0	0	0	0
Hafnia alvei	AIU74903.1	3857	-	25	1	0	0	0	0	0
Shigella dysenteriae	AHA63377	1442	-	25	0.9987	0.0012	0.0001	0	0	0
Citrobacter freundii	AHY14400	5630	6B, 4C	24	1.0001	0	0	0	0	0
Salmonella borgori	CCC32801.1	3197	-	24	1.0001	0	0	0	0	0

Campylobacter concisus	EAT97336	5080	4B, 3C	23	1.0001	0	0	0	0	0
Lactobacillus johnsonii	CAX65971	1423	4B, 4C	23	0.0065	0.9921	0.0007	0.0002	0.0002	0.0002
Escherichia fergusonii	CAQ88089	7222	7C	23	1	0	0	0	0	0
Enterobacter kobei	AOP86854	5403	-	23	1.0001	0	0	0	0	0
Providencia alcalifaciens	ATG15235.1	2520	9C	22	1	0	0	0	0	0
Parvimonas micra	AIZ36019	893	-	22	0.0278	0.9693	0.0021	0.0003	0.0002	0.0003
Finegoldia magna	BAG07467	2561	11B, 5C	21	0.0449	0.9468	0.007	0.0006	0.0004	0.0003
Lactobacillus acidophilus	AJP46937	1676	3B, 3C	20	1	0	0	0	0	0
Staphylococcus hyicus	AJC96154	1444	4C	15	0.0004	0.9989	0.0002	0.0002	0.0001	0.0001
Lactobacillus gasseri	ABJ59815	2457	-	13	0.9997	0.0003	0	0	0	0

The SignalP 6.0 server were used to predict the presence of signal peptides (SP)
SP: Signal peptide; LP: Lipoprotein

Table S2. ***Bap*****-coding genes**

Gene Bank ID	Bacteria	Position of *bap*-gene	Region of *bap* gene
AP008971.1	*Finegoldia magna* ATCC 29328	60332..68017	64994..68017
AP012281.1	*Lactococcus lactis subsp. lactis* IO-1	1416759..1420877	1416765..1417182
CP000792.2	*Campylobacter concisus* 13826	1952330..1967572	1952543..1955426
CP002491.1	*Enterococcus faecalis* 62	895380..898400	895572..897930
CP006262.1	*Escherichia coli* O145:H28 str. RM13516	508737..525725	513033..516392
CP006620.1	*Enterococcus faecium* Aus0085	2865948..2871146	2868597..2870969
CP007044.2	*Chania multitudinisentens* RB-25	2621577..2641064	2636825..2640776
CP007557.1	*Citrobacter freundii* CFNIH1	4834725..4851617	4848618..4851321
CP008747.1	*Staphylococcus hyicus* strain ATCC 11249	1432180..1436514	1432327..1433679
CP008876.1	*Terribacillus goriensis* strain MP602	2637246..2645765	2644266..2645678
CP010384.1	*Enterobacter hormaechei* subsp. *xiangfangensis* strain 34399	2175327..2193380	2187684..2189907
CP010432.1	*Lactobacillus acidophilus* strain FSI4	1640353..1645383	1642383..1644997
CP014229.1	*Desulfovibrio fairfieldensis* strain CCUG 45958	955636..975162	955753..958602
CP023536.1	*Providencia alcalifaciens* strain FDAARGOS_408	551626..559188	554181..557184
CU459141.1	*Acinetobacter baumannii* str. AYE	813035..837637	813113..815765
CU928158.2	*Escherichia fergusonii* ATCC 35469	544489..566157	547513..548688
FN298497.1	*Lactobacillus johnsonii* FI9785	23195..27466	23372..25234
JAGTJH_contig1y2	*Staphylococcus aureus* strain V329	635979..642809	642662..640350
UYIM01000001.1	*Clostridium indolis* strain NCTC11811	310911..317852	311571..315111
WMYK01000019.1	*Streptococcus salivarius* strain BIOML-A8	34778..39940	38672..39844

Gene Bank ID	Bacteria	Region of *csgA*
CP006262.1	*Escherichia coli* O145:H28 str. RM13516	1309918..1310374

Table S3. Metadata

Sample_Name	Sample	Case_status	Gender
MB23970	Feces	IBS	f
MB23972	Feces	IBS	f
MB23974	Feces	IBS	f
MB23976	Feces	IBS	f
MB23978	Feces	IBS	f
MB23980	Feces	IBS	m
MB23982	Feces	IBS	f
MB23984	Feces	IBS	m
MB23986	Feces	CT	f
MB23987	Feces	IBS	m
MB23988	Feces	IBS	m
MB23989	Feces	IBS	m
MB23991	Feces	CT	f
MB23992	Feces	IBS	f
MB23994	Feces	IBS	f
MB23996	Feces	IBS	f
MB23998	Feces	IBS	m
MB24000	Feces	IBS	m
MB24002	Feces	IBS	m
MB24003	Feces	IBS	m
MB24004	Feces	IBS	f
MB24006	Feces	IBS	f
MB24008	Feces	IBS	m
MB24010	Feces	IBS	f
MB24012	Feces	CT	m
MB24013	Feces	CT	f
MB24014	Feces	CT	f
MB24015	Feces	CT	m
MB24016	Feces	IBS	f
MB24355	Feces	IBS	f
MB24357	Feces	IBS	f
MB24359	Feces	CT	f
MB24360	Feces	CT	m
MB24361	Feces	IBS	m
MB24363	Feces	IBS	f
MB24365	Feces	IBS	f
MB24367	Feces	CT	m
MB24368	Feces	CT	f
MB24369	Feces	IBS	f
MB24371	Feces	IBS	f
MB24373	Feces	IBS	f
MB24375	Feces	IBS	f
MB24377	Feces	CT	m
MB24378	Feces	IBS	f
MB24380	Feces	IBS	f
MB24382	Feces	IBS	f
MB24384	Feces	CT	m
MB24385	Feces	CT	f
MB24386	Feces	CT	f

IBS: Intestinal bowel syndrome; CT: healthy control; f: feminine; m: masculine

Table S4 Amino acid stretches predicted to be amyloidogenic by at least four of the algorithms (black) or with the five algorithms (red)

Bacteria	Acc Nº	Name	Amino acids	Potential amyloid peptide*
Lactobacillus acidophilus	AJP46937	Bap_LA	345-354	DTAAIYAYSV
			655-662	SYYWYILN
			730-739	NQFLNNFNWW
			798-709	GLSYIQGVTWLD
Clostridium indolis	VDG65910	Bap_CI	540-547	NYQIFVRD
			888-893	TFNIID
Lactococcus lactis	BAL51295	Bap_LL	30-37	LLMTSVVI
			137-148	QIALINAIVSAG
			316-321	LDVNIL
			1346-1357	LGVIGTVILSLL
Lactobacillus johnsonii	CAX65971	Bap_LJ	27-37	SVLLGVTIFGI
			228-236	YKIVTIYAN
			326-331	FSTYIF
Staphylococcus hyicus	AJC96154	Bap_SH	70-77	GDIITVSW
			100-107	VVGQVLIN
			169-177	TGGVFYYKT
			188-196	VRWFLNINT
			261-266	INIFIP
			556-562	VVTINLL
			1420-1438	MQSWITWVVIGCIVLGIY F
Streptococcus salivarius	MTR00538.1	Bap_SS	243-250	SGYVYFTI
			583-590	TQLTYVGG
Enterococcus faecium	AGS76773	Esp_EFm	32-42	SVLVGVGLVLG
			226-232	YRIYLVS
			398-406	PVVIRYVLQL
			452-459	GTGYYYLQ
			690-696	AFSILDV
			710-716	TGIVTFT
			852-860	HKVIFTTGE
			937-943	KVIFTAG
			1063-1069	TVVVTYP
			1551-1557	GIVVVTY
			1705-1716	IFTISGGLILLG
Providencia alcalifaciens	ATG15235	Bap_PA	19-24	LDLIIT
			335-342	SVITLVIA
			670-676	QQFTFVP
			706-713	ITNINILS
			855-862	NYQIIITD
			963-970	YLLNNITV
			1431-1437	ATVTIII
			1450-1456	GHWLFTL
			1630-1638	FALVILSIS
Terribacillus goriensis	AIF67599	Bap_TG	816-824	STVVVINYD
			850-858	NATIIVTLA
			1604-1611	NDNFIFGL
			1615-1622	NSFIFLYL
			1990-1997	IIVVVTDP
			2075-2081	IVVVITD
			2161-2168	IIVVVTDP

			2246-2252	IVVVITD
			2331-2337	IIVVITD
			2416-2423	IIVVVTDP
			2501-2509	VIVVITDPA
			2586-2592	VIVVITD
			2671-2677	VIVVITD
			2722-2727	GVIVIL
Escherichia fergunsonii	CAQ90958	Bap_EFg	24-30	VTVVIYE
			248-254	WSVVQIV
			375-381	LAINTIA
			491-498	IVAQIISG
			670-679	PQVIINTFAG
			868-875	GAVLITIN
			1468-1475	AAITINTI
			1569-1574	TIIINT
			2006-2016	LTVVINTVTYA
			2067-2074	AAVAISIN
			2666-2673	TVAITINT
			2769-2775	VTINTIA
			2987-2991	DLIIS
			3067-3072	GVIINT
			3238-3243	GTVNIA
			3265-3272	AAVAITIN
			3567-3574	ILAINTIA
			4065-4070	VVTINT
			4135-4140	GTVTIN
			4401-4408	LTVTINSV
			4463-4469	AVAISIN
			4699-4704	VVSVVL
			5061-5068	TVAISINA
			5298-5305	LVVTIAGQ
			5398-5403	VTVLLN
			6048-6053	TWNYTV
			6428-6435	VEVVINGK
			6904-6911	VLVATQII
			6924-6931	ALVNFYGL
			6937-6943	YNAVLIK
Finegoldia magna	BAG08751	Bap_FM	38-45	MLGFIVLV
			137-142	IQAIVS
			480-486	TYYIIKN
			810-817	TETVTITV
			1058-1065	TETVTITV
			1182-1189	TETVTITV
			1306-1313	TETVTITV
			1430-1437	TETVTITV
			1554-1561	TETVTITV
			1678-1685	TETVTITV
			1802-1809	TETVTITV
			1926-1933	TETVTITV
Acinetobacter baumannii	CAM85746	Bap_AB	30-35	VVLVKV
			136-141	SDVIYQ
			238-243	VTVVIN
			411-415	TVVVN
			518-523	VTVVIN
			710-714	VTVVI
			902-907	VTVVIN
			1078-1082	TVVVN
			1422-1426	TVVVN

			1594-1598	TVVVN
			1766-1770	TVVVN
			1938-1942	TVVVN
			2110-2114	TVVVN
			2282-2286	TVVVN
			2454-2458	TVVVN
			2507-2512	AVVTID
			2626-2630	TVVVN
			2798-2802	TVVVN
			2970-2974	TVVVN
			3142-3146	TVVVN
			3314-3318	TVVVN
			3486-3490	TVVVN
			3658-3662	TVVVN
			3830-3834	TVVVN
			3883-3888	AVVTID
			4002-4006	TVVVN
			4174-4178	TVVVN
			4227-4232	AVVTID
			4262-4266	TVVVN
			4372-4378	NGVNYTV
			4477-4483	NGVNYTV
			4582-4588	NGVNYTV
			4687-4693	NGVNYTV
			4792-4798	NGVNYTV
			4897-4903	NGVNYTV
			5212-5218	NGVNYTV
			5485-5490	LITAIT
			5531-5537	NGVNYTV
			5846-5852	NGVNYTV
			6161-6167	NGVNYTV
			6266-6272	NGVNYTV
			6434-6440	LITAITV
			6543-6548	LITAIT
			6652-6657	LITAIT
			6697-6702	VNGVNY
			6866-6871	LITAIT
			6975-6980	LITAIT
			7084-7089	LITAIT
			7193-7198	LITAIT
			7471-7475	FTYSA
Desulfovibrio fairfieldensis	AMD89374	Bap_DF	423-430	SSTVITVR
			956-962	TVVTVSI
			1061-1069	SSILTVTIN
			1274-1281	NTLTVIVN
			1911-1919	NTLAVTING
			2652-2660	SNTLAVTIN
			3485-3492	DTFVVTVS
			3820-3826	TLIVTIN
			5005-5010	AVILED
			5988-5994	NVVYSLV
			6257-6261	DVFTY
Chania multitudinisentens	AHG22648	Bap_CM	96-101	LYAYVP
			429-436	GNVIIITD
			1208-1214	NQLTIVA
			1735-1741	NQLTIVA
			2262-2268	NQLTIVA

			2804-2810	YAITVLT
			2908-2913	FGIVVD
			3326-3333	YAITVITV
			3430-3435	FGIVVD
			3848-3854	YAITVLT
			3952-3957	FGIVVD
			4370-4376	YAITVIT
			4474-4479	FGIVVD
			4949-4953	ITIYN
			5409-5415	QAVLYSA
			5695-5699	LVVVN
			6364-6369	TFYLIN
			6444-6449	LNYLKV
Enterobacter hormaechei subsp. xiangfangensis	WP_022651163	Bap_ECl	8-12	DVIIR
			1108-1112	VTITI
			1163-1167	DVVVN
			1507-1512	VTIIFA
			1688-1693	GQALIV
			2286-2291	AQALIV
			2404-2408	VTVML
			2467-2473	PGVIINT
			3265-3271	TVAISIN
			3863-3868	AAVAIS
			4826-4832	TWQNVIV
			5064-5068	VRIID
			5226-5230	YVVIT
			5380-5386	YMVQLIN
			5403-5408	AVWYGA
			5850-5856	ATLVNIE
Escherichia coli O145:H28	AHG13161	Bap_ECo	12 17	TIIINT
			483-489	GTVNIIV
			510-517	AAVAISIN
			1081-1087	GTVNITV
			1211-1216	LVTINT
			1411-1416	LTISIV
			1509-1515	PGVIINT
			1547-1554	VIVTIGGN
			1647-1652	LTVVIN
			1681-1687	GTVNIAV
			1707-1715	LAVVAITIN
			1747-1753	ITVIFGG
			2011-2017	LAINTIA
			2507-2513	PVVTINT
			2905-2912	TAVAISIN
			3142-3147	VVSVVL
			3741-3749	LVVTIAGQQ
			3841-3846	VTVLLN
			4871-4876	VEVVIN
			5141-5145	NYYTL
			5347-5354	VLVATQII
			5366-5373	SALVNFYG
			5380-5386	YNAVLIK
Citrobacter freundii	AHY14400	Bap_CF	40-45	NVVVNV
			311-317	KIIVTID
			363-374	AIYANGVLLTTV
			463-467	ELYLN

			560-565	HIVITN
			659-664	VTIYID
			693-698	TAVAYA
			758-765	ATITVMVD
			994-1000	YIFYITA
			1060-1067	ISVQVIIN
			1093-1099	SISVIVT
			1255-1260	VQVIIN
			1399-1403	LVFTV
			1448-1453	VQVIIN
			1575-1581	TIVATVT
			1641-1646	VQVIIN
			1834-1839	VQVLIN
			1961-1967	TIVATVT
			2028-2034	VQVLING
			2176-2181	SFTIDT
			2224-2229	TITIAI
			2263-2267	YTITV
			2413-2418	PTFVIG
			2910-2915	YSVVVT
			3040-3045	SIVVNV
			3298-3305	YSVTVTIN
			3429-3434	SVVVTF
			3589-3595	TVFVVIN
			3684-3689	TLVVTV
			3816-3820	HIISV
			3882-3866	VTIYV
			4222-4227	TVVIDT
			4355-4359	ISILV
			4606-4612	STFTIVI
			4950-4956	GNWIYTP
			5323-5330	TTVTVTIN
Campylobacter concisus	WP_012140619	Bap_CC	1606-1612	VKFVEIT
			1944-1949	ITIYEI
			2000-2006	EVVVVNA
			2204-2210	VIKAVVT
			2317-2322	YRIVDL
			2611-2617	SVITIYE
			2669-2673	EVVVV
			2873-2879	VIKAVVT
			2986-2991	YRIVDL
			3280-3286	SVITIYE
			3338-3344	EVVVVNA
			3540-3546	VHVIKAV
			3655-3660	YRIVDL
			4103-4108	NVVAIA
			4370-4374	TIIAV
			4530-4536	GSVIIIK
			4784-4789	VKVSIV
			4822-4826	SVVAV
			4881-4885	ITFVV

Table S5 Amyloid domains exported at the C-DAG system

**Name**	
Bap_LA	LAGLITMWGTSGTNIVKINDPKYVNFQRTGSQPGSMLRLEGTTNSVSING<br>DGNAVTPVAQWDVGNTGNEPSYYWYILNETNQNNWGTNANGFTQKGE<br>TKPVNKDGEAKFLNSNGSVELAPNQSGSTASSYNNGTITESSDQTMYLNQ<br>FLNNFNWWTPQRIAMGSMLKDVATNAQEYKPEVKEITAGANDVLKDVD<br>PLEGITGLTDSNGNPVENGLSYIQGVTWLDS
Bap_CI	KLTNTWEVNFIRANGGLFGGAEILSQYTATNGKIELDDTVGNILNEAGNL<br>DNNKLNYQIFVRDSINNKIVRTSESS
Bap_LL	NLTIDFNQIALINAIVSAGDTFVAGVATILGNNPAASINLTQVTTQLNLLK<br>GIQNIGGGTFSTPTTLNGNSMLSAPLNDGMGAILSQNVTAILQNLRTAVNS<br>LTATGLAAPAANTALALIKPPLITAIDAVLVPLVNGTGGILDLLLNASALG<br>DTSITIPTKITAPTTIASNIDAKFVGSAVQTNLLDVNILSG
Bap_LJ	YKGTDGKYYKIVTIYANDYVYHTADIQANSTDTTAEDTKNNINISKEDLG<br>NGKTRWTVTFFPKKGLQNVGSRLSGLSSAKFGIALTNDYQILGNVDMDVI<br>SDPKQTFSTYIFKPGSNRATETTVQNPPAEVKFSFNPKTDVDENTGLINSK<br>TMPAYNNKYLQGPYYFTTATDIGKENLWQTYFKKWSLFGTVSNNAYDG<br>SPYLGTDHQLYFNSSKIKNKTGVDGAKEGNPVIYDTRDGVNGVFNSVNF<br>NQAMEFKSQGVTDQAQFSSYKISFTTQHTDSHEVDLAPGSKNQQFSGISA<br>NIYSYQNGGWNGF
Bap_SH	GDIITVSWPTTGNLKVEGYIKSLPLTVNEQVVGQVLINSDGATITFNQNVE<br>KLKDVSGFAEFEVQGRNLTNTTQQNTQNGSITSGTKSAELSVTKSEAGTG<br>GVFYYKTGDMQPSEPDYVRWFLNINTQKAYVIKDLTIHDQIQGGQKIDLS<br>TLNIQVEGSHPNNITGPTAIKEFQEKYPGSNLTVDESNNTINIFIPQGYA
Bap_SS1	NNVLHWTVLDNPGRKANAASGYVYFTIPKDSVGAPTNWQVVQTDKNG<br>KVVNTRNYWKDNDGSYLMGQNGRMDTYTGSEMTQRLTQLYKDIKNPA<br>IQGNEAAVAAQTAERSQALYTMTADNNAIRNVMWTITFDTP
Bap_SS2	PITVTKTQLTYVGGGVSGDNYTQLLVTESHLTVYPGDPVNVTIQAAGSPS<br>VEKFWIPNNPYLAKGLAYTNDSNGGFLDTAGSNTYRQRKAYYRGNVDM<br>TQPAGSSTATYAVRNKNGKVVTRNLTITVLETAKKYEPTPGGAKVEVAD<br>PNNISATEKAAIEKTVGDANTALPQGTTYVADEKGNVTITYPDKSVDKIA<br>AAYLVTP
Esp_EFm	SYSASAKGTPWYANYKIDERLVPYVAGIQMHMVQADKVTYDVSFESGK<br>KVADLAIERRKDHENYGVGSITDNDLTKLIDFANASPRPVVIRYVLQLTK<br>PLDEILEDMKATAQVEENKPFGEDFIFDSWLSDTNKKLIQNTYGTGYYYL<br>QDIDGDGNPDDKEESGDTNPYIG
Bap_PA1	EQQFTFVPLGNGASEAPLPTLSVMLDERSDSGVKEDYITNINILSFKGLAT<br>KGAKVSFDIGGKRYETTADHTTGKWEIKTETLQDGNNRYIVTATHPINGR<br>SLDVQGSVFIDSMLPISTIELTSETDTGTKGNFITSHHKPVFTGQGEVGCQV<br>TLTLNNETIKTTTDKHGQWSLQLSKELPKDFIGNYQIIITDAAGN
Bap_PA2	NATVTIIIDGKTHQVTADTAGHWLFTLPDKNALKDGHYDYQITATDILGQ<br>VSTQSLSGSIQIKTAPLVEVGLDSDSDSGVQNDNITNTQKPKLRGMTEPNA<br>DVRVIFDNQLAQPYQTKSDQNGHWFIEVTSELAEGHHDYIVTVNDVKQG<br>IRGEMAGEFTIDLTAPTELTAGVWHESEQVIKNTVLTNSATPTFKGQSEPF<br>ALVILSISKVGSHTIDI
Bap_TG	IDPVTDEDTNVSGDGEPGSTVVITLPDGSTIEAPVDEDGNWEADIPSQDPG<br>DEVIVVITDPAGNESDPVIVPVENADTEAPDTPS
Bap_EFg	VNSIEHGQALVITGSSSGLAAGAALTVVINTVTYAATVLADGTWSLGVPA<br>ADVGNWPAGTVDITVSGASSAGNPVTITHPVTVDLAAVAISINTVSGDDV<br>INAAEKGADLTLSGSTSGVEAGQTVTVTFGGKTYTATVAGD
Bap_FM	TITETVTITVQRDTDGDGIPDVDDDDDDNDGIKDEDDKNPKVADKLTLEA<br>TPKTQTVIEGQDIKDITAKVNKDGAVITNDQGLTVEGNTLKGKAPTVEW<br>KDDKHETEDVTVTITATKGEGEKAETITETVTITVQR
Bap_AB1	TVDTVPADLIGAITIPEDLNGDGILNADELGTDGSFNAQVALGPDALDGT<br>VVNVNGVNYTVTAADLANGYITAAIPVTGEGPVAIHAEAVDAQGNVDV<br>ADADVTV
Bap_AB2	TVPADLIGAITIPEDLNGDGILNADELGTDGSFNAQVALGPDALDGTVVN<br>VNGVNYTVTAADLANGYITAAIPVTGEGPVAIHAEAVDAQGNVDVADA<br>DVTVTVDTVPADLIGAITIPEDLNGDGILNADELGTDGSFNAQVALGPDA

	LDGTVVNVNGTNYTVTAADLANGYITAAIPVTGEGPVAIHAEAVDAQGN VDVADADVTVTVD
Bap_DF	TADIAEDAVGVSGALPPAVDPDLHDVASFIPQTDSAGLYGTLTLDASGNY TYILNNALPAVQGLGVGESLTDTFTFTASDGHGGTASSILTVTINGTNDAP TVTAAADDIAEDTVDISGTLPAPVDADTHDTVTFLPQTNTAGQYGTLNV DADGNYTYTLNNASPAVQGLGVGESLTDTFSFTVSDGHGGTATNTLTVTI NGTNDAP
Bap_CM1	GSVITVYDNGQVLGSVLTDNNGAWAFTPTTPLTEGEHSLTTTATDKAGN TSAPSDAFNLTTDYTPSLTGPEFLAITGVTDNVGNVQGNIASGGITDDSQP LISGIGTAGDTIMVYTADAAGNHLIGSATVQTDGTWSMTPELPLLEGNNQ LTIVAVDAAGNKTAPSTPSYDLNVDISIPTQPAITSVVDNVEPHTGALQKG DVTNDNTPTLSGSAEANSTVTVLDNGVAIGSVTA
Bap_CM2	ASPEYAITVITVPPPAPTITSVEDNAEPHTGALQKGDITNDNTPTLKGSALP GGIVTVFDNGTAIGSTTADNNGAWSFTPGTPLGEGNHNLAASVTDSIGQT SPTTGEFGIVVDTLPPAPVTDLVVTDDAGSVQGPLTAGSTTDDNTPTFSGK AEAGSVVTVLDNGKVIGSTTVDTNGN
Bap_ECl	AGQTVTVAFNGNQYTAQVQANGSWTLDVPAADMAGIADGSAAVTVTV SDKAGNPASAGASVLVDTTVPQITFDIVAGDDIVNIAEHGQALIVTGKVT GAQAGDVITLTLNGKDYTAMLDGAGSWSVGVPAADVGALANGDQTISA TVTDKAGNSTSATHAFDVSLTA
Bap_ECo	GLAAGAALTVVINGVTYGATVLTDGSWSVGVPAADVGNWPAGTVNIAV SGTNTAGTTTSITHPVTVDLAVVAITINTLSTDDVINAAEKGSDLQLSGTT SGVEAGQTITVIFGGKSYTTTVAADNSWGLTI
Bap_CF	NIDADVTKVQVIINGTAYDAVQSEGKWTFTAPELADGDYSITVQVTDDA GNVQTSTALAVTVDTSVAAPVIALSDDTGTAGDNQTNDTTPGFTVSTDP DAFSVMVSIDGGKPVAATKAADGQWHIDSSALTDGDHTIVATVTDL
Bap_CC	DSTPTLEGYAEPDSVITIYEISELNGEKTAIGTVTADNHGGWKFELPELGD GEYKYTTRAQDKAGNISDFSEVVVVNADLIAPSEPTITFV

Table S6. Purification of recombinant domains

	Amino-acids	pI	MW (kDa)	Expression vector	Primers
rBap_LA	585-810	4.39	24.5	pET46-Ek-LIC	5’-GACGACGACAAGATACTGGCAGGTCTGATTACC<br>5’-GAGGAGAAGCCCGGTTAGCTATCCAGCCAGGTAAC
rBap_CI	485-560	4.85	8.4	pET28a	5’-CCATGGGGAAACTGACCAATACCTGGG<br>5’-CTCGAGGCTGCTTTCGCTGGTACGAA
rBap_LL	130-323	4.31	19.4	pET46-Ek-LIC	5’-GACGACGACAAGATAAATCTGACCATTGATTTTA<br>5’-GAGGAGAAGCCCGGTTAATTAGTGGTGATGGTGATGA
rBap_SS2*	577-780	8.61	22	pET28a	5’-CCATGGGGCCGATTACCGTTACCAAAACA<br>5’- CTCGAGCGGTGTAACCAGATATGCTG
rBap_EFm	313-481	4.89	20.4	pET28a	5’- CCATGGGGTATTCAGCTTCCGCAAAGGGC<br>5’- CTCGAGCCCGATATATGGATTCGTGTC
rBap_TG	2448-2532	2.76	8.6	pET28a	5’-CCATGGGGATTGATCCGGTTACCGATG<br>5’-CTCGAGGCTCGGTGTATCCGGAGCTT
rBap_CM2	3322-3500	3.68	23.6	pET46-Ek-LIC	5’-GACGACGACAAGATAGGTAGCGTTATTACCGTTT<br>5’-GAGGAGAAGCCCGGTTATGCGGTAACACTACCAATT
rBap_EFg	1982-2122	3.95	13.5	pET28a	5’-CCATGGGGGTTAATAGCATTGAACATGGT<br>5’-CTCGAGATCACCGGCAACGGTGGC
rBap_FM	1304-1439	4.20	14.7	pET46-Ek-LIC	5’-GACGACGACAAGATAACCATTACCGAAACCGTT<br>5’-GAGGAGAAGCCCGGTTAGCGCTGCACGGTAATCGT
rBap_ECl	1602-1768	3.88	16.4	pET28a	5’-CCATGGGGGCAGGTCAGACCGTTACCGTT<br>5’-CTCGAGTGCGGTCAGGCTAACATCAAA
rBap_CF	1440-1583	3.7	14.7	pET28a	5’- CCATGGGGAATATTGATGCAGATGTTACCAAA<br>5’- CTCGAGCAGATCGGTAACGGTTGCAAC
rBap_LJ	74-580	6.49	34.6	pET28a	5’-CCATGGGGACCCCACAATCTTCACCAGAG<br>5’-CTCGAGCCGCAAAACATTAATTTGAGC
rBap_SH	70-270	4.80	21.9	pET46-Ek-LIC	5’-GACGACGACAAGATAGGCGATATTATCACCGTTAGC<br>5’-GAGGAGAAGCCCGGTTAGCATAGCCCTGCGGGATA
rBap_PA1*	669-866	5.72	22.8	pET28a	5’- CCATGGGGGAACAGCAGTTTACCTTTGTTCCG<br>5’- CTCGAGATTGCCTGCTGCATCGGT
rBap_ECo	1640-1770	3.9	12.8	pET28a	5’-CCATGGGGGGTTTAGCAGCCGGTGCA<br>5’-CTCGAGAATGGTCAGACCCCAGCTATT

*For *S. salivarius* and *P. alcalifaciens* we chose domain Bap_SS2 and Bap_PA1 respectively because their expression at the C-DAG system provided higher CR binding than Bap_SS1 and Bap_PA2
Domains shown in grey could not be produced in a soluble form

Table S7. Association between BAP-genes and PD adjusting variables by age, sex, stool collection and total sequence count

Gene Bank ID	Gene	Region of *bap* gene	PD Nº	PD summary stats	NHC Nº	NHC summary stats	Total Nº	P	OR [95%CI]	P*	OR [95%CI]*
AP008971.1	*bap_FM*	64994..68017	490	40 (8%)	234	9 (4%)	724	3.90E-02	2.2 [1-5.3]	1.52E-02	2.6 [1.3-6.1]
CP002491.1	*esp*	895572..897930	490	44 (9%)	234	7 (3%)	724	2.80E-03	3.2 [1.4-8.5]	8.98E-03	3.1 [1.4-7.7]
CP006262.1	*bap_ECo*	513033..516392	490	136 (28%)	234	44 (19%)	724	1.00E-02	1.7 [1.1-2.5]	1.56E-02	1.6 [1.1-2.5]
CP006620.1	*bap_EFm*	2868597..2870969	490	36 (7%)	234	4 (2%)	724	1.40E-03	4.6 [1.6-17.8]	2.07E-03	5.4 [2.1-18.7]
CU928158.2	*bap_EFg*	547513..548688	490	93 (19%)	234	26 (11%)	724	7.30E-03	1.9 [1.2-3.1]	1.67E-02	1.8 [1.1-3.0]
FN298497.1	*bap_LJ*	23372..25234	490	83 (17%)	234	17 (7%)	724	3.20E-04	2.2 [1-5.3]	2.57E-03	2.4 [1.4-4.4]
CP006262.1	*csgA*	1309918..1310374	490	42 (9%)	234	6 (3%)	724	2.10E-03	3.2 [1.4-8.5]	4.38E-03	3.7 [1.6-10.0]

*adjusted by sex, age, collection method and total sequences; PD: Persons Parkinson's disease; NHC: Neurologically healthy control subjects; P: P-value. OR [95%CI]: odds ratio and 95% confidence interval of the difference between PD and NHC. Data were analyzed by binary logistic regression using the glm() function with binominal distribution in R.

Table S8. Analysis of confounding factors in detail. MaAsLin2 was used for testing.

Variable	Gene Bank ID	Gene	Region of *bap* gene	Odds Ratio	95% Confidence Interval		P	Significance levels2
Case status	AP008971.1	*bap_FM*	64994..68017	2.63	1,26	6.07	0.01518	*
Sex	AP008971.1	*bap_FM*	64994..68017	0.79	0,43	1.47	0.45798	
Age (standardized)	AP008971.1	*bap_FM*	64994..68017	0.98	0,95	1.02	0.27475	
Stool collection method	AP008971.1	*bap_FM*	64994..68017	10.49	1,32	67.96	0.0131	*
Total sequence count (standardized)	AP008971.1	*bap_FM*	64994..68017	1.00	1.00	1.00	0.41298	
Case status	CP002491.1	*esp*	895572..897930	4.66	1.26	30.27	0.04559	**
Sex	CP002491.1	*esp*	895572..897930	0.79	0.31	2.08	0.62903	
Age (standardized)	CP002491.1	*esp*	895572..897930	1.02	0.96	1.08	0.56223	
Stool collection method	*CP002491.1*	*esp*	895572..897930	10.27	0.49	84.91	0.04963	
Total sequence count (standardized)	CP002491.1	*esp*	895572..897930	1.00	1.00	1.00	0.36819	
Case status	CP006262.1	*bap_ECo*	513033..516392	1.80	1.04	3.27	0.04392	*
Sex	CP006262.1	*bap_ECo*	513033..516392	1.16	0.71	1.90	0.5637	
Age (standardized)	CP006262.1	*bap_ECo*	513033..516392	1.00	0.97	1.03	0.95525	
Stool collection method	CP006262.1	*bap_ECo*	513033..516392	1.86	0.09	13.09	0.58497	
Total sequence count (standardized)	CP006262.1	*bap_ECo*	513033..516392	1.00	1.00	1.00	0.08448	
Case status	CP006620.1	*bap_EFm*	2868597..2870969	8.31	1.62	152.48	0.04316	**
Sex	CP006620.1	*bap_EFm*	2868597..2870969	1.07	0.41	3.05	0.89654	
Age (standardized)	CP006620.1	*bap_EFm*	2868597..2870969	1.03	0.97	1.09	0.36621	
Stool collection method	*CP006620.1*	*bap_EFm*	2868597..2870969	11.03	0.51	101.04	0.04831	
Total sequence count (standardized)	CP006620.1	*bap_EFm*	2868597..2870969	1.00	1.00	1.00	0.23578	
Case status	CU928158.2	*bap_EFg*	547513..548688	2.31	1.27	4.49	0.008656	*
Sex	CU928158.2	*bap_EFg*	547513..548688	1.16	0.70	1.93	0.570465	
Age (standardized)	CU928158.2	*bap_EFg*	547513..548688	1.00	0.98	1.03	0.779936	
Stool collection method	CU928158.2	*bap_EFg*	547513..548688	2.00	0.10	14.45	0.543237	
Total sequence count (standardized)	*CU928158.2*	*bap_EFg*	547513..548688	1.00	1.00	1.00	0.030433	**

Case status	*FN298497.1*	*bap_LJ*	23372..25234	4.36	1.82	12.95	0.00269	**
Sex	FN298497.1	*bap_LJ*	23372..25234	0.81	0.44	1.50	0.494269	
Age (standardized)	*FN298497.1*	*bap_LJ*	23372..25234	1.05	1.01	1.10	0.008711	
Stool collection method	FN298497.1	*bap_LJ*	23372..25234	4.13	0.20	33.68	0.23104	
Total sequence count (standardized)	FN298497.1	*bap_LJ*	23372..25234	1.00	1.00	1.00	0.938717	

Linear regression was performed using MaAsLin2 tes (Microbiome Multivariable Association with Linear Models). SE: standard error of Beta; P: the uncorrected, two-sided P-value reported by MaAsLin2 or ANCOM-BC; FDR: false discovery rate, i.e., multiple-testing corrected significance q-value, calculated using the Benjamini-Hochberg method; FC: fold change in PD compared to NHC of the relative abundance (MaAsLin2) of a species, calculated by taking exponent of Beta using base 2 (MaAsLin2); FC lower and upper: lower and upper bound of the 95% confidence interval for the FC; NT: not tested due to prevalence filter of 5% of subjects.

Table S9. Statistical analysis of the relative abundance (RPKM) of BAP genes using MaAsLin

Gene Bank ID	Gene	Region of *bap* gene	Beta	SE	P	FDR	FC	FC lower	FC upper	N.not .0
CP002491.1	*esp*	895572..897930	0,25	0,08	0,003	0,011	1,188	1,023	1,352	51
CP006620.1	*bap_EFm*	2868597..2870969	0,33	0,11	0,002	0,011	1,261	1,046	1,476	40
CP006262.1	*bap_ECo*	513033..516392	0,59	0,24	0,015	0,032	1,510	1,033	1,987	388
CU928158.2	*bap_EFg*	547513..548688	0,50	0,21	0,016	0,032	1,414	1,009	1,819	119
AP008971.1	*bap_FM*	64994..68017	0,14	0,08	0,095	0,166	1,103	0,937	1,268	49
FN298497.1	*bap_LJ*	23372..25234	0,90	0,22	0,000	0,001	1,862	1,430	2,293	100
CP006262.1	*csgA*	1309918..1310374	0,98	0,40	0,014	0,032	1,979	1,193	2,764	180

Linear regression was performed using MaAsLin2 tes (Microbiome Multivariable Association with Linear Models). SE: standard error of Beta; P: the uncorrected, two-sided P-value reported by MaAsLin2 or ANCOM-BC; FDR: false discovery rate, i.e., multiple-testing corrected significance q-value, calculated using the Benjamini-Hochberg method; FC: fold change in PD compared to NHC of the relative abundance (MaAsLin2) of a species, calculated by taking exponent of Beta using base 2 (MaAsLin2); FC lower and upper: lower and upper bound of the 95% confidence interval for the FC; NT: not tested due to prevalence filter of 5% of subjects.

Table S10 Strains and plasmids

MIC	Strains and plasmids		Reference
		**CDAG-system**	
1881	*E. coli* VS39	Curli-deficient *E. coli* MC4100 (*csgA-, csgB-, csgC-*) transformed with pVS76 (cat PlacUV5 *csgG*, pACYC184 ori; produces CsgG)	[36]
2153	*E. coli* VS39-cured	*E. coli* VS39 cured of pVS76 plasmid	This study
2405	VS39 CsgA	*E. coli* VS39 complemented with pEXPORT:*csgA* from *E. coli*	[36]
1869	VS39 Bap_B	*E. coli* VS39 complemented with pEXPORT:*bap_B,* expression vector for C-DAG system, including amino acids from 361 to 819 of Bap_B from *S. aureus*	[15]
1870	VS39 Bap_A	*E. coli* VS39 complemented with pEXPORT:*bap_A,* expression vector for C-DAG system including amino acids from 49 to 361 of Bap_A from *S. aureus*	[15]
1868	VS39 Esp_N	*E. coli* VS39 complemented with pEXPORT:*esp_N,* expression vector for C-DAG system, including amino acids from 50 to 743 of Esp_N from *E. faecalis*	[16]
2000	VS39 Esp_RC	*E. coli* VS39 complemented with pEXPORT:*esp_RC* from *E. faecalis*	[16]
2192	VS39 Bap_LA	*E. coli* VS39 complemented with pEXPORT:*bap_LA,* expression vector for C-DAG system, including amino acids from 585 to 810 of Bap_like from *L. acidophilus*	This study
2234	VS39 Bap_CI	*E. coli* VS39 complemented with pEXPORT:*bap_CI*, expression vector for C-DAG system, including amino acids from 485 to 560 of Bap_like from *C. indolis*	This study
2237	VS39 Bap_LL	*E. coli* VS39 complemented with pEXPORT:*bap_LL*, expression vector for C-DAG system, including amino acids from 130 to 323 of Bap_like from *L. lactis*	This study
2133	VS39 Bap_LJ	*E. coli* VS39 complemented with pEXPORT:*bap_LJ,* expression vector for C-DAG system, including amino acids from 230 to 530 of Bap_like from *L. johnsonii*	This study
2191	VS39 Bap_SH	*E. coli* VS39 complemented with pEXPORT:*bap_SH,* expression vector for C-DAG system, including amino acids from 70 to 270 of Bap_like from *S. hyicus*	This study
2118	VS39 Bap_SS1	*E. coli* VS39 complemented with pEXPORT:*bap_SS1,* expression vector for C-DAG system, including amino acids from 224 to 358 of Bap_like from *S. salivarius*	This study
2119	VS39 Bap_SS2	*E. coli* VS39 complemented with pEXPORT:*bap_SS2,* expression vector for C-DAG system, including amino acids from 577 to 780 of Bap_like from *S. salivarius*	This study
2597	VS39 Esp_EFm	*E. coli* VS39 complemented with pEXPORT:*bap_EFm,* expression vector for C-DAG system, including amino acids from 313 to 481 of Esp from *E. faecium*	This study
2235	VS39 Bap_PA1	*E. coli* VS39 complemented with pEXPORT:*bap_PA1,* expression vector for C-DAG system, including amino acids from 669 to 866 of Bap_like from *P. alcalifaciens*	This study
2236	VS39 Bap_PA2	*E. coli* VS39 complemented with pEXPORT:*bap_PA2,* expression vector for C-DAG system, including amino acids from 1428 to 1647 of Bap_like from *P. alcalifaciens*	This study
2194	VS39 Bap_TG	*E. coli* VS39 complemented with pEXPORT:*bap_TG.* expression vector for C-DAG system, including amino acids from 2448 to 2531 of Bap_like from *T. goriensis*	This study

2120	VS39 Bap_FM	*E. coli* VS39 complemented with pEXPORT:*bap_FM1,* expression vector for C-DAG system, including amino acids from 1304 to 1439 of Bap_like from *F. magna*	This study
1864	VS39 Bap_AB1	*E. coli* VS39 complemented with pEXPORT:*bap_AB1,* expression vector for C-DAG system, including amino acids from 1817 to 1988 of Bap_like from *A. baumannii*	This study
1866	VS39 Bap_AB2	*E. coli* VS39 complemented with pEXPORT:*bap_AB2,* expression vector for C-DAG system, including amino acids from 5795 to 6004 of Bap_like from *A. baumannii*	This study
1903	VS39 Bap_DF	*E. coli* VS39 complemented with pEXPORT:*bap_DF,* expression vector for C-DAG system, including amino acids from 975 to 1181 of Bap_like from *D. fairfieldensis*	This study
2195	VS39 Bap_CM1	*E. coli* VS39 complemented with pEXPORT:*bap_CM1,* expression vector for C-DAG system, including amino acids from 1060 to 1294 of Bap_like from *C. multitudinisentens*	This study
2196	VS39 Bap_CM2	*E. coli* VS39 complemented with pEXPORT:*bap_CM2,* expression vector for C-DAG system, including amino acids from 3322 to 3500 of Bap_like from *C. multitudinisentens*	This study
2197	VS39 Bap_EFg	*E. coli* VS39 complemented with pEXPORT:*bap_EFg,* expression vector for C-DAG system, including amino acids from 1982 to 2122 of Bap_like from *E. fergusonii*	This study
2796	VS39 Bap_ECl	*E. coli* VS39 complemented with pEXPORT:*bap_EH,* expression vector for C-DAG system, including amino acids from 1602 to 1768 of Bap_like from *E. hormaechei*	This study
2797	VS39 Bap_ECo	*E. coli* VS39 complemented with pEXPORT:*bap_EC,* expression vector for C-DAG system, including amino acids from 1640 to 1770 of Bap_like from *E. coli O145:H28*	This study
2798	VS39 Bap_CF	*E. coli* VS39 complemented with pEXPORT:*bap_CF,* expression vector for C-DAG system, including amino acids from 1440 to 1583 of Bap_like from *C. freundii*	This study
2799	VS39 Bap_CC	*E. coli* VS39 complemented with pEXPORT:*bap_CC,* expression vector for C-DAG system, including amino acids from 1929 to 2019 of Bap_like from *C. concisus*	This study
**Heterologous complementation with amyloid domains**			
1888	*S. aureus* Δbap	*S. aureus* V329 *bap-*	[95]
2184	Δbap Empty	*S. aureus* Δbap containing pCN51, a *E. coli*-*S. aureus* shuttle vector with a cadmium inducible promoter (Pcad)	This study
2183	Δbap Bap_B	*S. aureus* Δbap containing pCN51:*bap_B*, plasmid for expression of Bap_B (amino acids from 361 to 819) fused to the R-domain of ClfA, under the control of the Pcad promoter	[15]
1889	Δbap Esp_N	*S. aureus* Δbap containing pCN51:*esp_N*, plasmid for expression of Esp_N (amino acids from 50 to 743) fused to the R-domain of ClfA, under the control of the Pcad promoter	[16]
2249	Δbap Bap_LA	*S. aureus* Δbap containing pCN51:*bap*_LA, plasmid for expression of N-terminal of domain of Bap_LA (amino acids from 585 to 810) fused to the R-domain of ClfA, under the control of the Pcad promoter.	This study
2582	Δbap Bap_CI	*S. aureus* Δbap containing pCN51:*bap*_CI, plasmid for expression of N-terminal of domain of Bap_CI (amino acids from 485 to 560) fused to the R-domain of ClfA, under the control of the Pcad promoter	This study

2584	Δbap Bap_LL	*S. aureus* Δbap containing pCN51:*bap*_LL, Plasmid for expression of N-terminal of domain of Bap_LL (amino acids from 130 to 323) fused to the R-domain of ClfA, under the control of the Pcad promoter	This study
2583	Δbap Bap_LJ	*S. aureus* Δbap containing pCN51:*bap*_LJ, plasmid for expression of N-terminal of domain of Bap_LJ (amino acids from 221 to 411) fused to the R-domain of ClfA, under the control of the Pcad promoter	This study
2827	Δbap Bap_SH	*S. aureus* Δbap containing pCN51:*bap*_SH, plasmid for expression of N-terminal of domain of Bap_SH (amino acids from 71 to 206) fused to the R-domain of ClfA, under the control of the Pcad promoter	This study
2185	Δbap Bap_SS1	*S. aureus* Δbap containing pCN51:*bap*_SS1, plasmid for expression of N-terminal of domain of Bap_SS (amino acids from 224 to 358) fused to the R-domain of ClfA, under the control of the Pcad promoter	This study
2739	Δbap Bap_SS2	*S. aureus* Δbap containing pCN51:*bap*_SS2, plasmid for expression of N-terminal of domain of Bap_SS (amino acids from 577 to 780) fused to the R-domain of ClfA, under the control of the Pcad promoter	This study
2706	Δbap Esp_EFm	*S. aureus* Δbap containing pCN51:*bap*_EFm, plasmid for expression of N-terminal of domain of Esp_EFm (amino acids from 313 to 481) fused to the R-domain of ClfA, under the control of the Pcad promoter	This study
2826	Δbap Bap_PA1	*S. aureus* Δbap containing pCN51:*bap*_PA1, plasmid for expression of N-terminal of domain of Bap_PA (amino acids from 669 to 866) fused to the R-domain of ClfA, under the control of the Pcad promoter	This study
Primers for Bap_LA amplification		5’-GCGGAAGTACAAAATAGTGCTGCAGGTCTGATTACCATGTGG<br>5’-GGTACCGCTATCCAGCCAGGTAACACC	
Primers for Bap_CI amplification		5’-GCGGAAGTACAAAATAGTGCTAAACTGACCAATACCTGG<br>5’-CCGGGTACCGCTGCTTTCGCTGGTACGA	
Primers for Bap_LL amplification		5’GCGGAAGTACAAAATAGTGCTAATCTGACCATTGATTTTAATCAG<br>5’GGTACCACCTGACAGAATATTAACATCCAG	
Primers for Bap_LJ amplification		5’GCGGAAGTACAAAATAGTGCTAAAGGCACCGATGGCAAA<br>5’GGTACCAACGGTGCCAAACAGTGA	
Primers for Bap_SH amplification		5’GCGGAAGTACAAAATAGTGCTGATATTATCACCGTTAGCTGG<br>5`GGTACCGGTCAGATCTTTAATCACGTAGG	
Primers for Bap_SS1 amplification		5’GCGGAAGTACAAAATAGTGCTAATAATGTTCTGCATTGGACC<br>5’GGTACCCGGTGTATCAAAGGTGATGGT	
Primers for Bap_SS2 amplification		5’GCGGAAGTACAAAATAGTGCTCCGATTACCGTTACCAAAACA<br>5’GGTACCCGGTGTAACCAGATATGCTG	
Primers for Bap_EFm amplification		5’GCGGAAGTACAAAATAGTGCTCGATTAGTTCCATATGTA<br>5’GGTACCCCCGATATATGGATTCGT	
Primers for Bap_PA1 amplification		5’GCGGAAGTACAAAATAGTGCTGAACAGCAGTTTACCTTTG<br>5’GGTACCATTGCCTGCTGCATCGGTG	
		**Expression system of recombinant amyloids**	
1897	*E. coli* BL21(DE3)	*E. coli* used for protein expression using the T7 expression system (F¯ *omp*T *gal dcm lon hsd*SB(rB- mB-) λ(DE3 **lacI lacUV5-T7 gene 1 ind1 sam7 nin5*])	
1884	BL21 BapB	BL21(DE3) transformed with pET46-eK-LIC:Bap_B, plasmid for expression of Bap_B (amino acids from 361 to 819)	[15]
1959	BL21 Esp_N	BL21(DE3) transformed with pET46-eK-LIC:Esp_N, plasmid for expression of Esp_N (amino acids from 50 to 743)	[16]

2247	BL21 Bap_LA	BL21(DE3) transformed with pET46-eK-LIC:Bap_LA, plasmid for expression of Bap_LA (amino acids from 585 to 810)	This study
2639	BL21 Bap_CI	BL21(DE3) transformed with pET28a:Bap_CI, plasmid for expression of Bap_CI (amino acids from 485 to 560)	This study
2436	BL21 Bap_LL	BL21(DE3) transformed with pET46-eK-LIC:Bap_LL, plasmid for expression of Bap_LL (amino acids from 130 to 323)	This study
2951	BL21 Bap_LJ4	BL21(DE3) transformed with pET28a:Bap_LJ, plasmid for expression of Bap_LJ (amino acids from 74 to 580)	This study
2853	BL21 Bap_SH	BL21(DE3) transformed with pET46-eK-LIC:Bap_SH, plasmid for expression of Bap_SH (amino acids from 70 to 270)	This study
2224	BL21 Bap_SS2	BL21(DE3) transformed with pET46-eK-LIC:Bap_SS2, plasmid for expression of Bap_SS2 (amino acids from 577 to 780)	This study
2640	BL21 Bap_TG	BL21(DE3) transformed with pET28a:Bap_TG, plasmid for expression of Bap_TG (amino acids from 2448 to 2532)	This study
2435	BL21 Bap_CM2	BL21(DE3) transformed with pET46-eK-LIC:Bap_CM2, plasmid for expression of Bap_CM2 (amino acids from 3322 to 3500)	This study
2698	BL21 Bap_EFg	BL21(DE3) transformed with pET28a:Bap_EFg, plasmid for expression of Bap_EFg (amino acids from 1982 to 2122)	This study
2222	BL21 Bap_FM	BL21(DE3) transformed with pET46-eK-LIC:Bap_FM, Pplasmid for expression of Bap_FM (amino acids from 1304 to 1439)	This study
2810	BL21 Bap_EFm	BL21(DE3) transformed with pET28a:Esp_EFm, plasmid for expression of Esp_EF (amino acids from 313 to 481)	This study
2809	BL21 Bap_PA1	BL21(DE3) transformed with pET28a:Bap_PA1, Plasmid for expression of Bap_PA (amino acids from 669 to 866)	This study
2870	BL21 Bap_ECl	BL21(DE3) transformed with pET28a:Bap_ECl plasmid for expression of Bap_ECl (amino acids from 1602 to 1768)	This study
2852	BL21 Bap_CF	BL21(DE3) transformed with pET28a:Bap_CF plasmid for expression of Bap_CF (amino acids from 1440 to 1583)	This study
2854	BL21 Bap_ECo	BL21(DE3) transformed with pET28a:Bap_ECo plasmid for expression of Bap_ECo (amino acids from 1640 to 1770)	This study
**Other purposes**			
2167	*E. coli* OP50	Uracil auxotroph *E. coli* used for feeding nematodes	
