# Supplementary material for: Gut microbiota produces biofilm-associated amyloids with potential for neurodegeneration
Source: Nat Commun. 2024 May 16;15:4150. doi: 10.1038/s41467-024-48309-x (PMC11099085; doi:10.1038/s41467-024-48309-x)
Supplement: Supplementary file 3 — Reporting Summary [file 41467_2024_48309_MOESM3_ESM.pdf]

nature portfolio

Corresponding author(s): Jaione Valle

Last updated by author(s): 2024/04/23

Reporting Summary

Nature Portfolio wishes to improve the reproducibility of the work that we publish. This form provides structure for consistency and transparency in reporting. For further information on Nature Portfolio policies, see our Editorial Policies and the Editorial Policy Checklist.

Statistics

For all statistical analyses, confirm that the following items are present in the figure legend, table legend, main text, or Methods section.

n/a	Confirmed	
☐	☒	The exact sample size (*n*) for each experimental group/condition, given as a discrete number and unit of measurement
☒	☐	A statement on whether measurements were taken from distinct samples or whether the same sample was measured repeatedly
☐	☒	The statistical test(s) used AND whether they are one- or two-sided<br>*Only common tests should be described solely by name; describe more complex techniques in the Methods section.*
☒	☐	A description of all covariates tested
☒	☐	A description of any assumptions or corrections, such as tests of normality and adjustment for multiple comparisons
☐	☒	A full description of the statistical parameters including central tendency (e.g. means) or other basic estimates (e.g. regression coefficient) AND variation (e.g. standard deviation) or associated estimates of uncertainty (e.g. confidence intervals)
☐	☒	For null hypothesis testing, the test statistic (e.g. *F*, *t*, *r*) with confidence intervals, effect sizes, degrees of freedom and *P* value noted<br>*Give P values as exact values whenever suitable.*
☒	☐	For Bayesian analysis, information on the choice of priors and Markov chain Monte Carlo settings
☒	☐	For hierarchical and complex designs, identification of the appropriate level for tests and full reporting of outcomes
☒	☐	Estimates of effect sizes (e.g. Cohen's *d*, Pearson's *r*), indicating how they were calculated

Our web collection on statistics for biologists contains articles on many of the points above.

Software and code

Policy information about availability of computer code

Data collection	DNA-seq data were collected using commercial softwares from Illumina. Fluorescence and absorbance measures were taken using a BioTek Synergy H1 microplate reader. Microscopy images were taken using commercial softwares from Leica. Negative staining images were taken using a JEOL 1011 and JEM 1400 transmission electron microscopes. FTIR spectra were acquired using a Bruker Tensor 27 FTIR (Bruker Optics, USA) supplied with a Specac Golden Gate MKII ATR accessory. Light scattering was measured using a Jasco FP-8200 fluorescence spectrophotometer (Jasco Corporation, Japan). Quantitative PCR were performed using Applied BiosystemsTM QuantStudio 5 machine and StepOneTM Real-Time PCR system (Applied Biosystems). For locomotive analysis of C. elegans swimming worms movements were collected with a Zeiss Stemi 508 stereo microscope and Zeiss Axiocam 208 color camera.
Data analysis	For DNA-seq data analysis, the host DNA was filtered out by mapping against host reference genome using KneadData v0.74. Trimmomatic v0.38 was used to remove Illumina sequencing adapter and trim low quality end bases. To assess quality of raw data and trimmed data, FASTQC v0.11.6 was performed. Centrifuge v1.0.4 was performed for taxonomy assignment of trimmed data with primary assignments set as 1. The count and portion of the reads assigned to each taxon number were calculated at each taxon ranks (Kingdom, Phylum, Class, Order, Family, Genus, Species and Subspecies). Normalized abundance of taxon at level of species was calculated as: Abundance = Ra/Rt *G; where Ra: Read count assigned to the taxon; Rt: Total read count; G: Average genome size of the taxon. After calculating abundance, relative abundance which is ratio of abundance, compared with total abundance sum was calculated. The abundance of BAP was calculated as reads per kilobase per million mapped reads (RPKM).<br>For Fluorescent microscopy analysis, image processing was performed using Leica DMi8 fluorescence microscope and Hamamatsu ORCA Flash 4.0 LT camera. Image processing was performed with Icy software. For transmission electron microscope analysis, samples were scanned using a CCD GATAN ES1000W Erlangshen camera<br>For FTIR data were acquired and normalized using the OPUS MIR Tensor 27 software (Bruker Optics, USA), with the Min/Max normalization

method. Data was deconvoluted by automated peak fitting using the Peak Fit software. The resulting area, amplitude and central values of the fitted bands were plotted.

For the metagenomic data reanalysis we performed a quality control checks on the raw sequencing reads using FastQC v0.12.1 to filter low quality and adapter-contaminated reads. We used FastQ Screen v0.15.3 to check the presence of other organism's contaminations within the samples. We proceeded to remove a Nextera transposase adapter using Cutadapt v4.34 with –trim-n, --quality-cutoff 25 and –a CTGTCTCTTATA –A CTGTCTCTTATA to clean forward and reverse reads from that adapter. We employed Centrifuge v1.0.4 for a general taxonomic profiling and functional annotation. The high-quality 150 nt pairend reads were aligned against a custom database, consisting of a reference genome collection comprising different bacteria that contained BAP gene region using BWA v0.7.17-r1188 (default parameters). The alignment process enabled us to determine the proportion of BAP presence in the samples by examining their positional information with SAMtools v1.157. Reads that contained less than 70 matches in their sequence were removed. We employed Pysam to assess the alignment quality of the reads against the reference database.

For locomotive analysis of C. elegans, recorded worms were tracked with the WrmTrck plugin from ImageJ (https://www.phage.dk/plugins/wrmtrck.html) to measure the body bends per minute (BBPM).

For manuscripts utilizing custom algorithms or software that are central to the research but not yet described in published literature, software must be made available to editors and reviewers. We strongly encourage code deposition in a community repository (e.g. GitHub). See the Nature Portfolio guidelines for submitting code & software for further information.

Data

Policy information about availability of data

All manuscripts must include a data availability statement. This statement should provide the following information, where applicable:

- Accession codes, unique identifiers, or web links for publicly available datasets
- A description of any restrictions on data availability
- For clinical datasets or third party data, please ensure that the statement adheres to our policy

Sequencing data and metadata are available online through European Nucleotide Archive (ENA) with the study accession PRJEB66343.

We used the raw shotgun sequencing data obtained from individuals diagnosed with PD, along with Neurologically Healthy Controls (NHC) available at the NCBI Sequence Read Archive (SRA) repository under the BioProject ID PRJNA834801

Research involving human participants, their data, or biological material

Policy information about studies with human participants or human data. See also policy information about sex, gender (identity/presentation), and sexual orientation and race, ethnicity and racism.

Reporting on sex and gender	Sex and gender were considered in the study design
Reporting on race, ethnicity, or other socially relevant groupings	Samples and data from patients included in the study were provided by the Biobank of the University of Navarra and were processed following standard operating procedures approved by the Ethical and Scientific Committees. Samples were kept at -80 °C until further processing. 35 individuals with irritable bowel syndrome (IBS) and 14 control were enrolled at the study.
Population characteristics	Participants included 14 healthy controls and 35 IBS patients. The average age of the participants was 45 years. The oldest was 82 years old and the youngest was 17 years old. Thirty-two women and 17 men were included in the study.
Recruitment	The patients were selected from the Digestive Department of the Clínica Universidad de Navarra. Those patients who consulted with symptoms suggestive of IBS have been screened for IBS according to the Rome IV diagnostic criteria. Once the diagnosis was established, patients were asked to enter the study. During a specific visit, the objective of the study was explained to them, as well as the conditions for their participation and they were asked to sign a written informed consent form.
Ethics oversight	The protocol 2020.153 was aproved by the Comité de Ética de la Investigación de la Universidad de Navarra

Note that full information on the approval of the study protocol must also be provided in the manuscript.

Field-specific reporting

Please select the one below that is the best fit for your research. If you are not sure, read the appropriate sections before making your selection.

☒ Life sciences ☐ Behavioural & social sciences ☐ Ecological, evolutionary & environmental sciences

For a reference copy of the document with all sections, see nature.com/documents/nr-reporting-summary-flat.pdf

Life sciences study design

All studies must disclose on these points even when the disclosure is negative.

Sample size	Sample-size calculation was not performed since it was not possible to predict the magnitude of the variation between animals (mice or C. elegans) for a particular parameter based on our current knowledge. However, considering previous studies that determine the effect of amyloids (alpha-Syn-PFF or curli), at least 12 animals (mice or worms) per group were included in all in vivo experiments. This allowed us to

detect statistically significant differences among groups. In neuron culture experiments the aggregation of a-Syn was determined in at least 20 cells. This allowed us to detect statistically significant differences among groups. For molecular biology and biochemical tests, at least two replicates were performed, where each replicate was assembled and measured independently. This allowed us to detect statistically significant differences among groups. For the patients' data, all available patients and samples that matched the inclusion criteria were included.

Data exclusions	No data were excluded
Replication	Experiments were performed at least two times. When experiments were repeated, they yielded comparable results.
Randomization	In animal experiments, all animals were randomly distributed in the cages and before any procedure, the cages were randomized to each group by a person not involved in the study.
Blinding	Mice experiments were blinded and the investigators responsible for data collection and analysis were blinded.

Reporting for specific materials, systems and methods

We require information from authors about some types of materials, experimental systems and methods used in many studies. Here, indicate whether each material, system or method listed is relevant to your study. If you are not sure if a list item applies to your research, read the appropriate section before selecting a response.

Materials & experimental systems

n/a	Involved in the study	
☐	☒	Antibodies
☐	☒	Eukaryotic cell lines
☒	☐	Palaeontology and archaeology
☐	☒	Animals and other organisms
☒	☐	Clinical data
☒	☐	Dual use research of concern
☒	☐	Plants

Methods

n/a	Involved in the study	
☒	☐	ChIP-seq
☒	☐	Flow cytometry
☒	☐	MRI-based neuroimaging

Antibodies

Antibodies used

Primary:
Rabbit Polyclonal anti-amyloid fibrils (OC). StressMarq; ref: SPC-507S. Dilution for WB 1:1.000
Rabbit Polyclonal anti Amyloid Oligomers (A11). StressMarq; ref: SPC-506. Dilution for WB 1:1.000
Rabbit Polyclonal anti-BapB. Ref: PMID:27327765. Dilution for WB dilution 1:5.000. Dilution for IF 1:200
Chicken Polyclonal anti-EspN. Ref: PMID:33424817. Dilution for WB 1:5.000.
Rabbit Polyclonal anti-BapLL. Ref: this study. Dilution for WB 1:5.000.
Rabbit Polyclonal anti-BapLA. Ref: this study. Dilution for WB 1:5.000.
Mouse monoclonal anti-GFP JL-8. Takara; ref: 632381 Dilution for WB 1:5.000.
Rabbit monoclonal anti--Syn (PS129) (EP1536Y). Abcam; ref: ab51253 Dilution for WB 1:1.000. Dilution for IF 1:200
Mouse monoclonal anti--Syn LB509. Invitrogen; ref: 180215. Dilution for WB 1:1.000. Dilution for IF 1:200
Rabbit Polyclonal anti-LAMP2A. Invitrogen; ref: 51-2200. Dilution for WB 1:5.000.
Rabbit Polyclonal anti-Beta Actin. Abcam; ref: ab8227. Dilution for WB 1:5.000.
Rabbit Polyclonal anti-Tyrosine Hydroxylase. Abcam; ref: ab112. Dilution for WB 1:2.000.
Rabbit Polyclonal anti-NeuN. Abcam; ref: ab104225. Dilution for WB 1:5.000
Rabbit Polyclonal anti-Iba1 (for Immunocytochemistry). Wako; ref: 019–19741. Dilution for IF 1:500
Mouse monoclonal anti-GFAP. Sigma; ref: MAB360. Dilution for IF 1:1.000
Mouse monoclonal anti-polyhistidine. Sigma; ref: H1029
Secondary:
Goat anti-chicken-HRP. Abcam; ref: Ab97135 Dilution for WB 1:20.000
Goat anti-rabbit-IgG-HRP. Invitrogen; ref: 31460 Dilution for WB 1:5.000
Goat anti-mouse-IgG-HRP Life technologies; ref: A16072 Dilution for WB 1:5.000
Goat anti-rabbit-ALEXA 488 Invitrogen; ref: A11008. Dilution for IF 1:200
Goat anti-mouse-ALEXA 568 Invitrogen; ref: A11031. Dilution for IF 1:200
Goat-anti-Mouse IgG (H&L) (10nm) AURION (ref: 810.022)

Validation

- Primary antibody anti-amyloid fibrils (OC) (StressMarq; ref: SPC-507S) was validated by manufacturer (https://www.stressmarq.com/products/antibodies/polyclonal-antibodies/amyloid-fibrils-oc-antibody-spc-507/) to preferentially recognize Abeta42 fibrils by dot blot and wester-blot and plaque amyloids in tissue from Alzheimer's Disease brain by IF and Immunohistochemistry.
- Rabbit Polyclonal anti Amyloid Oligomers (A11) (StressMarq; ref: SPC-506) was validated by manufacturer (https://www.stressmarq.com/products/antibodies/polyclonal-antibodies/amyloid-oligomers-a11-antibody-spc-506/) to preferentially recognize Abeta42 prefibrillar oligomers by dot blot and wester-blot and plaque amyloids in tissue from Alzheimer's Disease brain by IF.

- Rabbit Polyclonal anti-BapB was validated by Taglialegna et al 2016; PMID:27327765 to recognize Bap surface protein and Bap-mediated amyloids of Staphylococcus aureus by wester-blot and IF. Antibody is routinely used in our laboratory.
- Chicken Polyclonal anti-EspN was validated by Taglialegna et al 2020; PMID:33424817 to recognize Esp surface protein and Esp-mediated amyloids of Enterococcus faecalis by wester-blot and IF. Demonstrated reactivity with Esp+ strain and lack of reactivity with Esp- strain by western-blot an immunofluorescence microscopy. Antibody is routinely used in our laboratory.
- Rabbit Polyclonal anti-BapLL Ref: this study. Anti-BapLL recognizes BapLL surface protein of Lactococcus lactis by wester-blot and IF. Demonstrated reactivity using BapLL positive strain and lack of reactivity with BapLL negative strain by western-blot an immunofluorescence microscopy.
- Rabbit Polyclonal anti-BapLA Ref: this study. Anti-BapLA recognizes BapLA surface protein of Lactococcus lactis by wester-blot and IF. Demonstrated reactivity using BapLA positive strain and lack of reactivity with BapLA negative strain by western-blot an immunofluorescence microscopy.
- Mouse monoclonal anti-GFP JL-8 (Takara; ref: 632381) was validated by manufacturer demonstrating reactivity by Western blot analysis using lysate from a HEK 293 cell line stably expressing AcGFP1 (chromeextension://efaidnbmnnnibpcajpcglclefindmkaj/https://www.takarabio.com/documents/Certificate%20of%20Analysis/632380/632380-632381-070313.pdf)
- Rabbit monoclonal anti--Syn (PS129) EP1536Y (Abcam; ref: ab51253). Antibody ab51253 shows high specificity for pS129-αSyn detection. Clone EP1536Y robustly detected endogenous pS129-α-syn in highly soluble protein fractions from the mouse brain. Clone EP1536Y was able to detect endogenous pS129-α-syn via immunoblot. Occasionally may stain unknown high-molecular weight protein that is not α-Syn (100 KDa) in immunoblots (PMID: 32156613) (PMID: 29888794).
- Mouse monoclonal anti--Syn LB509 (Invitrogen; ref: 180215) was validated by manufacturer. LB509 reacts with α-synuclein but not SMsynuclein. Reactivity has not been observed with α-synuclein. Positive tissue: Parkinson's disease tissue. (https://www.thermofisher.com/antibody/product/Alpha-synuclein-Antibody-clone-LB509-Monoclonal/180215)
- Rabbit Polyclonal anti-LAMP2A (Invitrogen; ref: 51-2200) was validated by manufacturer. Western blot analysis was performed on whole cell extracts (30 µg lysate) of C2C12, NIH/3T3 and PC-12. A ~110 kDa band corresponding to LAMP2 was observed across the cell lines tested. (https://www.thermofisher.com/antibody/product/LAMP-2A-Antibody-clone-AMC2-Polyclonal/51-2200)
- Rabbit Polyclonal anti-Beta Actin (Abcam; ref: ab8227) was validated by manufacturer to detect human B-actin by IHC-P, IHC-FrFl, ELISA, Dot-blot, Western-blot. (https://www.abcam.com/en-es/products/primary-antibodies/alpha-synuclein-phospho-s129-antibody-ep1536y-ab51253#)
- Rabbit Polyclonal anti-Tyrosine Hydroxylase (Abcam; ref: ab112) was validated by manufacturer to detect rat Th and mammal Th (predicted) by IHC-P and Western-blot. (https://www.abcam.com/en-es/products/primary-antibodies/tyrosine-hydroxylase-antibody-neuronal-marker-ab112#).
- Rabbit Polyclonal anti-NeuN (Abcam; ref: ab104225) was validated by manufacturer to detect mouse and human RBFOX3 by IHC-P, IHC-FrFl, IHC-FoFr, Western-blot. (https://www.abcam.com/en-es/products/primary-antibodies/neun-antibody-neuronal-marker-ab104225#).
- Rabbit Polyclonal anti-Iba1 (Wako; ref: 019-19741) was validated by manufacturer to detect ionized calcium binding adaptor molecule 1 (IBA1) of iCell Microglia by immunofluorescence (https://www.fujifilmcdi.com/anti-iba1-polyclonal-antibody-019-19741)
- Mouse monoclonal anti-GFAP (Sigma; ref: MAB360) was validated by manufacturer. Reactivity with other species has not been determined. In Western blotting of extracts from a human glioma cell line (U33CG/343MG), MAB360 recognises a band at approximately 51 kDa corresponding to GFAP (Debus, 1983). By immunohistochemistry it recognises astrocytes and Bergmann glial cells, glioma and glial cell-derived tumours. It does not show cross-reactivity with vimentin.
- Mouse monoclonal anti-polyhistidine (Sigma; ref: H1029) was validated by manufacturer. The antibody recognises native or denatured reduced forms of synthetic polyhistidine and polyhistidine-tagged fusion proteins. The antibody preferentially recognises the N-terminally labelled fusion protein and is reactive with fusion proteins expressed by the prokaryotic expression vectors PET, pRSET and pTrc. https://www.sigmaaldrich.com/ES/es/product/sigma/h1029#product-documentation

Eukaryotic cell lines

Policy information about cell lines and Sex and Gender in Research

Cell line source(s)	SH-SY5Y cells from ECACC. Species: human. Tissue of origin: neural. Disease: neuroblastoma. The clone over-expressing WT alpha-synuclein was generated form the ECACC cell line.
Authentication	Cell lines were validated and authenticated by western blot, sequencing and immunohistochemistry
Mycoplasma contamination	Cell line tested was negative for mycoplasma contamination
Commonly misidentified lines (See ICLAC register)	No misidentified cell lines were used

Animals and other research organisms

Policy information about studies involving animals; ARRIVE guidelines recommended for reporting animal research, and Sex and Gender in Research

Laboratory animals	C57BL6/C3H F1 (Charles River) Mice 8-9 week old, males.<br>CD1 6 week old, females<br>C. elegans NL5901 pkls2386[Punc-54::-Syn::YFP + unc-119(+)]
Wild animals	No
Reporting on sex	The findings reported apply to both sex, however in order to reduce the number of C57BL6/C3H F1 mice used, the study was only performed in males.

Field-collected samples	No
Ethics oversight	Mice studies were approved by the Ethics Committee on Animal Experimentation of the Center of Biomedical Research of La Rioja (CIBIR, permit number LAE-04) and the Comité de Ética y Bioseguridad del IdAB-CSIC (Ref. number 1357/2022)

Note that full information on the approval of the study protocol must also be provided in the manuscript.

Plants

Seed stocks	*Report on the source of all seed stocks or other plant material used. If applicable, state the seed stock centre and catalogue number. If plant specimens were collected from the field, describe the collection location, date and sampling procedures.*
Novel plant genotypes	*Describe the methods by which all novel plant genotypes were produced. This includes those generated by transgenic approaches, gene editing, chemical/radiation-based mutagenesis and hybridization. For transgenic lines, describe the transformation method, the number of independent lines analyzed and the generation upon which experiments were performed. For gene-edited lines, describe the editor used, the endogenous sequence targeted for editing, the targeting guide RNA sequence (if applicable) and how the editor was applied.*
Authentication	*Describe any authentication procedures for each seed stock used or novel genotype generated. Describe any experiments used to assess the effect of a mutation and, where applicable, how potential secondary effects (e.g. second site T-DNA insertions, mosiacism, off-target gene editing) were examined.*
